# Interrelations of vegetation growth and water scarcity in Iran revealed by satellite time series

**DOI:** 10.1038/s41598-022-24712-6

**Published:** 2022-12-01

**Authors:** Robert Behling, Sigrid Roessner, Saskia Foerster, Peyman Saemian, Mohammad J. Tourian, Tanja C. Portele, Christof Lorenz

**Affiliations:** 1 Remote Sensing and Geoinformatics Section, Helmholtz Centre Potsdam GFZ German Research Centre for Geosciences, Potsdam, Germany; 2 Institute of Geodesy, University of Stuttgart, Stuttgart, Germany; 3 Karlsruhe Institute of Technology (KIT), Campus Alpin, Institute of Meteorology and Climate Research - Atmospheric Environmental Research (IMK-IFU), Garmisch-Partenkirchen, Germany

**Keywords:** Sustainability, Environmental impact

## Abstract

Iran has experienced a drastic increase in water scarcity in the last decades. The main driver has been the substantial unsustainable water consumption of the agricultural sector. This study quantifies the spatiotemporal dynamics of Iran’s hydrometeorological water availability, land cover, and vegetation growth and evaluates their interrelations with a special focus on agricultural vegetation developments. It analyzes globally available reanalysis climate data and satellite time series data and products, allowing a country-wide investigation of recent 20+ years at detailed spatial and temporal scales. The results reveal a wide-spread agricultural expansion (27,000 km$^2$) and a significant cultivation intensification (48,000 km$^2$). At the same time, we observe a substantial decline in total water storage that is not represented by a decrease of meteorological water input, confirming an unsustainable use of groundwater mainly for agricultural irrigation. As consequence of water scarcity, we identify agricultural areas with a loss or reduction of vegetation growth (10,000 km$^2$), especially in irrigated agricultural areas under (hyper-)arid conditions. In Iran’s natural biomes, the results show declining trends in vegetation growth and land cover degradation from sparse vegetation to barren land in 40,000 km$^2$, mainly along the western plains and foothills of the Zagros Mountains, and at the same time wide-spread greening trends, particularly in regions of higher altitudes. Overall, the findings provide detailed insights in vegetation-related causes and consequences of Iran’s anthropogenic drought and can support sustainable management plans for Iran or other semi-arid regions worldwide, often facing similar conditions.

## Introduction

Under the expected growth of population and climate projections, a large part of the world’s population is going to face conditions of increasing water scarcity and food insecurity^1–6^. The higher food demand being a consequence of growing global population requires an increase in food production, which can either be met by expanding the area under cultivation or intensifying the use of the already existing agricultural land^7^. At global scale, it is assumed that these options have the potential to fulfil the growing global food needs^3,7^. However, regions under unsuitable food production conditions (e.g. unsuited climate, soil, and relief) might have to increase their food production beyond a sustainable stage or have to rely on food imports to ensure food security for the population.

Iran is a prominent example for such conditions. The country has been facing a rapid population growth^8^ accompanied by unfavorable political conditions preventing extensive food imports, and thus the country has significantly increased local food production during the past 30+ years^9–12^, although large parts of Iran are unsuited or of limited suitability for agricultural purposes^7^. This development has led to a high and ever increasing water demand towards a very unsustainable use of renewable water sources^9,12–14^. At present, Iran uses more than 80% of its total renewable freshwater resources, while 40% is considered being the limit to ensure environmental sustainability^9,12^.

Major parts of Iran experience very limited water availability. More than 90% of the country are under arid or semi-arid conditions and 75% of the precipitation is received during the winter season when it is not needed for the agricultural sector^9^. Mesgaran et al. (2017)^7^ rate almost 80% of Iran’s land as (very) poorly suited or unsuited for cropping. Since thousands of years people had to cope with this situation and the Persians once were known for their advanced and sustainable (adapted to local conditions) water management, e.g. by building subsurface qanats to efficiently transfer water from the mountains to the adjacent plains and valleys^9,15,16^. These historical developments had their origin in the foothills of the Zagros Mountains, which are known for an emerging agriculture thousands of years ago^16–18^.

However, in the last decades the rapid socioeconomic development^19^ and climatic change towards drier conditions have changed this situation completely. Iran’s population has grown rapidly from approx. 20 million in 1960 to more than 80 million people today^8,20,21^, with 70% living in urban areas (27% in the 1950s), which creates a high pressure on regional available water resources. From the 1960s on, Iran started a big modernization project to allow higher water consumption rates meeting the steadily increasing population. These modernization measures include the replacement of traditional sustainable irrigation techniques (e.g. qanats)^15^ with electric pumps for groundwater exploitation, the construction of hundreds of dams with more to come in the future, and the realization of large water transfer projects across major drainage divides^9,13,22^.

Furthermore, Iran has promoted the paradigm of food self-sufficiency after the Islamic Revolution in 1979, due to food shortages during Iran-Iraq war and thereafter decade-long embargo policies against the country^7,9,12^. To support the agricultural sector, the government has heavily subsidized agricultural water and energy use, which led to low prices raising no need to increase agricultural production efficiency^9,23^. As a result, the agricultural sector is responsible for approx. 90% of the annual water consumption in Iran and thus drives the country’s large and unsustainable water use^10,13,18^. Approximately 50% of the water used for agriculture comes from tapping underground aquifers^13,22^ making Iran one of the top groundwater miners in the world^22,24–26^ and resulting in a severe decline of groundwater levels throughout the country^9,12,22,27,28^.

Recent climate change and future climate projections tend towards warmer and overall drier conditions in Iran, adding further pressure on available water resources^26,28^. The last decades showed a clear trend in rising temperature, whereas often no significant trends in annual precipitation could be observed^29,30^. Nevertheless trends towards less consecutive wet days^31^, an increasing share of intense precipitation events on the annual budget^29,31^, an increase of green water deficit^32,33^, and a decrease of precipitation in the warm season^34^ were identified, which overall have led to less plant-available water. Using integrative drought indices (such as AI, IDM, RDI, SDI), several studies showed an increase of dry conditions and more frequent drought events^21,35–39^. Climate models confirm the recent trends and project further increase in temperature and an overall decrease of precipitation leading to extended dry periods interrupted by intermittent heavy rainfalls, increasing the potential of flash floods and putting more pressure on water availability for agricultural use in particular and the vegetation cover in general^26,32,33,40–42^.

Consequences of increasing water scarcity during the last decades are e.g. drying up of lakes and rivers^43–45^, declining groundwater resources^13,22^, land subsidence^27,46–49^, water contamination^50,51^, water supply rationing and disruptions^52–54^, formation of soil salinization^55^ and sandstorms^56^, forced human migration, agricultural losses, and widespread ecosystem damages^9,26,37,44,57,58^.

In the present study we analyze the interrelationships between vegetation growth, land cover dynamics, and continuously increasing water scarcity in Iran on a country-wide scale with the goal of assessing vegetation growth dynamics as well as evaluating and quantifying related agricultural developments in high spatiotemporal detail. So far, for Iran only province based cumulative statistics about the agricultural development exist in terms of total numbers on annual crop yield and harvested area that gets reported by the Ministry of Agriculture-Jahad^59,60^. However, a systematic and spatiotemporal explicit analysis of the development of agricultural land use and related vegetation growth has been missing on a country-wide scale. Moreover, consequences of the increasing water scarcity to natural vegetated biomes have not been analyzed yet for whole Iran. The presented study has been carried out in the frame of the BMBF (Federal Ministry of Education and Research of Germany) funded SaWaM project (Seasonal Water resources Management in semi-arid regions: regionalized global data and transfer to practice) within the GRoW initiative (Global Resource Water) aiming at utilizing and adapting global datasets for the needs of regional water resource management in semi-arid regions.

We systematically analyze vegetation growth dynamics between 2001 and 2019 using multi-temporal satellite remote sensing data (MODIS), which allow investigation of vegetation conditions in high spatial and temporal detail at a country-wide scale. The derived spatiotemporal vegetation growth dynamics are evaluated against the dynamics of hydrometeorological conditions (using ERA5-Land, GRACE(-FO)) and land cover changes (using ESA-CCI-LC), as major factors controlling vegetation growth changes under Iran’s highly diverse natural conditions. Our analysis of global scale reanalysis climate model data and globally available satellite remote sensing data and its derived products enables:

- Quantification of spatiotemporal dynamics of vegetation growth, hydrometeorological water availability, land cover, and their interrelation,

with special focus on the

- Evaluation of agricultural developments, i.e. gains and losses of agricultural areas, intensification and degradation of agricultural land usage, and irrigation intensity of agricultural areas by analyzing vegetation growth against meteorological water availability.

## Results

### Hydrometeorological dynamics

**Figure 1:**
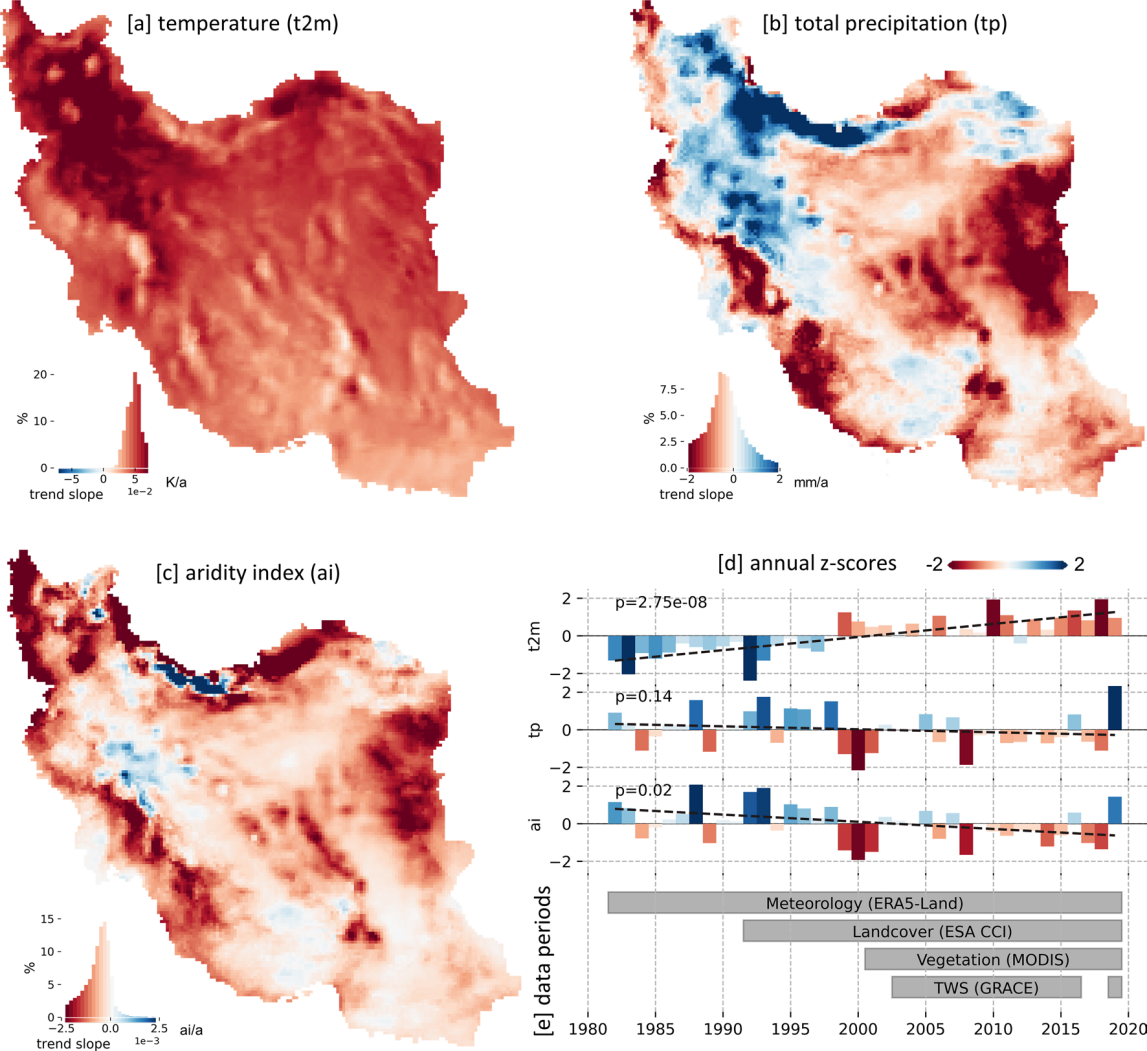
Trends and annual variations of meteorological input parameters from ERA5-Land between 1982 and 2019. (**a**)–(**c**): Trend slope of the gridded data at 0.1 degree. (**d**): Annual variations to the long-term mean (Z-scores of Iran wide averages) with trend line (dashed line) along with associated p-values of the trend using Mann-Kendall test. (**e**): Periods of the analyzed parameter of this study. General note: The colorscale is chosen to represent dry/warm conditions as red, thus the colormap of *t*2*m* is inverted compared to *tp* and *ai*. Maps were created using Python 3.9 (https://www.python.org/).

Iran has experienced a country-wide average warming of approx. 1.7 K between 1982 and 2019 (trend line of air temperature at 2m (*t*2*m*) in Fig. 1d). Temperature (*t*2*m*) has risen all over the country with the highest increase in northwest Iran and lowest in southeast Iran (Fig. 1a). In contrast, total precipitation (*tp*) shows no significant Iran wide trend (Fig. 1d) and mainly statistically insignificant pixel-based trends (Fig. S7) with spatial variations to drier and wetter conditions. The aridity index (*ai*), being an integrative measure of dryness as the ratio between water input (*tp*) and water loss (potential evaporation), shows a significant Iran wide trend towards drier conditions (Fig. 1d) and a relatively homogenous distribution of dry trends (Fig. 1c), which are however mainly statistically insignificant (Fig. S7). Figure 1e depicts the meteorological dynamics in relation to the availability periods of the global data products used in this study. It shows that for Iran, the analyzed period of vegetation dynamics (covered by MODIS data) coincides with a pronounced severe dry period between 1999 and 2001 forming the onset of a 20-year warm and dry period (compared to 1980–2000) ending with a very wet year in 2019, which was characterized by extensive flooding^61–64^. Thus, the available MODIS based vegetation growth observations almost exclusively comprise a continuous warm and dry period that started in the year 1999.

**Figure 2:**
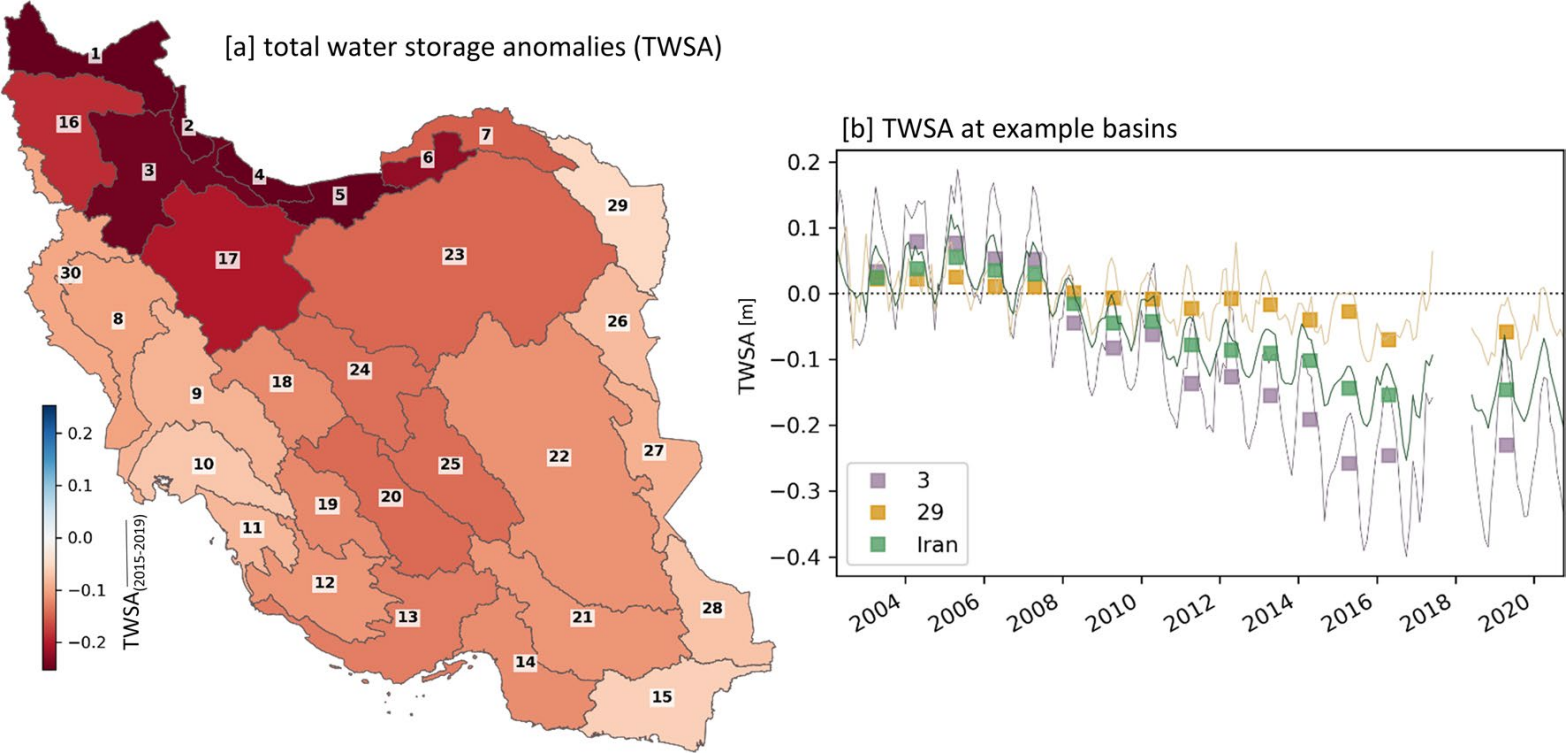
Total water storage anomalies (*TWSA*) observed by GRACE(-FO) (Methods). (**a**): Map showing the average annual *TWSA* for 2015/2016/2019 per basin as an indicator for the most recent situation. (**b**): *TWSA* for entire Iran and two selected basins; no. 3 with stronger and no. 29 with weaker decreasing *TWS* (line: monthly *TWSA*; rectangle: annual *TWSA*, representing an average of monthly data for a water year). Map was created using Python 3.9 (https://www.python.org/).

The total water storage (*TWS*) measured by the GRACE(-FO) satellite missions has decreased over Iran between 2004 and 2019. The strongest decrease occurred in the wetter northern and northwestern basins of Iran (Fig. 2a). The main loss occurred between 2008 and 2015 and since then the *TWS* has been remaining on a lower level (Fig. 2b). The nationwide decline of *TWS* is not refelected by a decrease in freshwater input, since no significant negative trend of *tp* could be observed since 1982 (Fig. 1d) or since the GRACE(-FO) data availability since 2003 (Fig. S8d). The basins with the highest loss of *TWS* in the northwest of Iran show even rather positive trends of *tp* (Fig. 1b, Fig. S8b).

### Land cover changes

**Figure 3:**
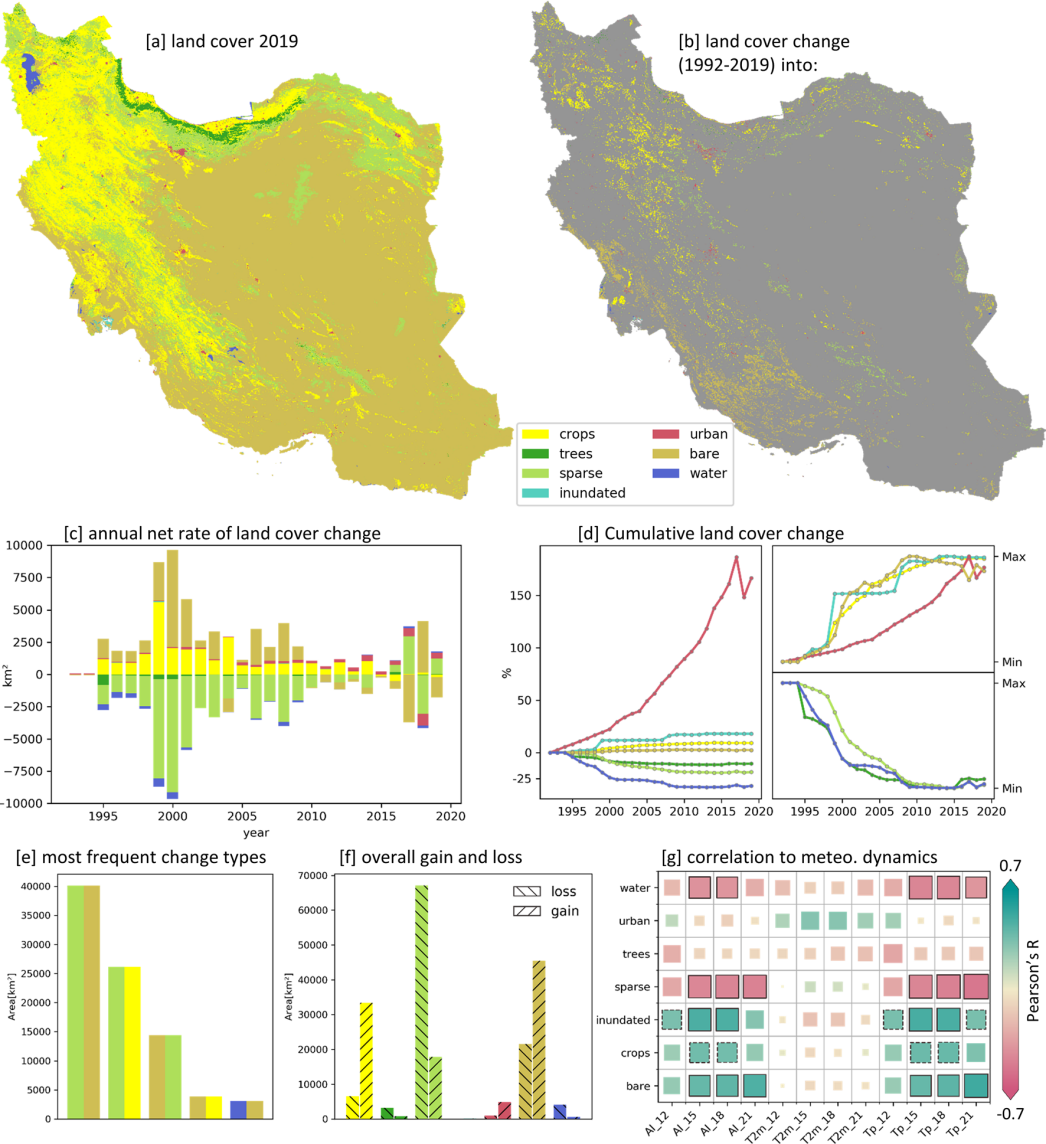
Land cover dynamics of Iran between 1992 and 2019. (**a**): Land cover map of 2019. (**b**): Map of the land cover that the pixel changed into from 1992–2019. (**c**): Annual net rate (annual gain - annual loss). (**d**): Cumulative land cover change. Left: change in percent related to 1992. Right: normalized change curves to long-term min and max, to highlight change intensities over time. (**e**): Five most frequent transition types. Double bars represent land cover transition from (left) to (right) land cover class. (**f**): Overall changes (gains and losses) during 1992 and 2019. (**g**): Pearson correlation between land cover annual net rate (Fig. 3c) and meteorological parameters (*ai*, *t*2*m*, *tp*) aggregated over the water year corresponding to land cover year (*_12) and over a prolonged time period including previous year’s conditions (e.g., *_15 = *_12 and previous 3 month). Maps were created using Python 3.9 (https://www.python.org/).

According to the adapted ESA-CCI land cover data sets (Methods), in 2019 the three spatially dominating land cover classes of Iran are bare land, cropland, and sparse vegetation with 62%, 20%, and 13% areal coverage, respectively (Fig. 3a). Comparing the annual land cover data sets for Iran between 1992 and 2019 reveals substantial changes (Fig. 3, Table S2). Urban areas have largely increased (+4037 km$^{2}$ / +166%) reflecting the enormous population growth and urbanization process of the last decades^8,20^. Agricultural areas also have expanded substantially (+26,771 km$^{2}$ / +9%), showing the need to turn non-agricultural land into cropland. Moreover, an increase of bare land (+24,033 km$^{2}$ / +2%) and decrease of sparse vegetation (−49,245 km$^{2}$ / −19%) could be observed, which is most likely the result of a combination of anthropogenically induced land use changes and the trend towards drier meteorological conditions prevailing during the last decades. Figure 3e shows that the change of sparse vegetation into bare ground (40,110 km$^{2}$) is the spatially most dominating land cover change type implying a large-scale decrease of plant-available water. The annual net change rate of bare ground and sparse vegetation correlates significantly with the water availability (in terms of *tp* and *ai* shown in Fig. 3g), suggesting that dry conditions result in an increase of bare ground and a decrease of sparse vegetation and vice versa in case of wetter conditions. This relationship also gets evident in the large decrease of sparse vegetation and large increase of bare ground (Fig. 3c) during the severe drought period between 1999 to 2001 (Fig. 1), showing that such pronounced dry periods can have a large impact on vegetation cover in such vulnerable (semi-)arid environments.

### Vegetation growth dynamics

#### Long-term spatial distribution of vegetation growth

**Figure 4:**
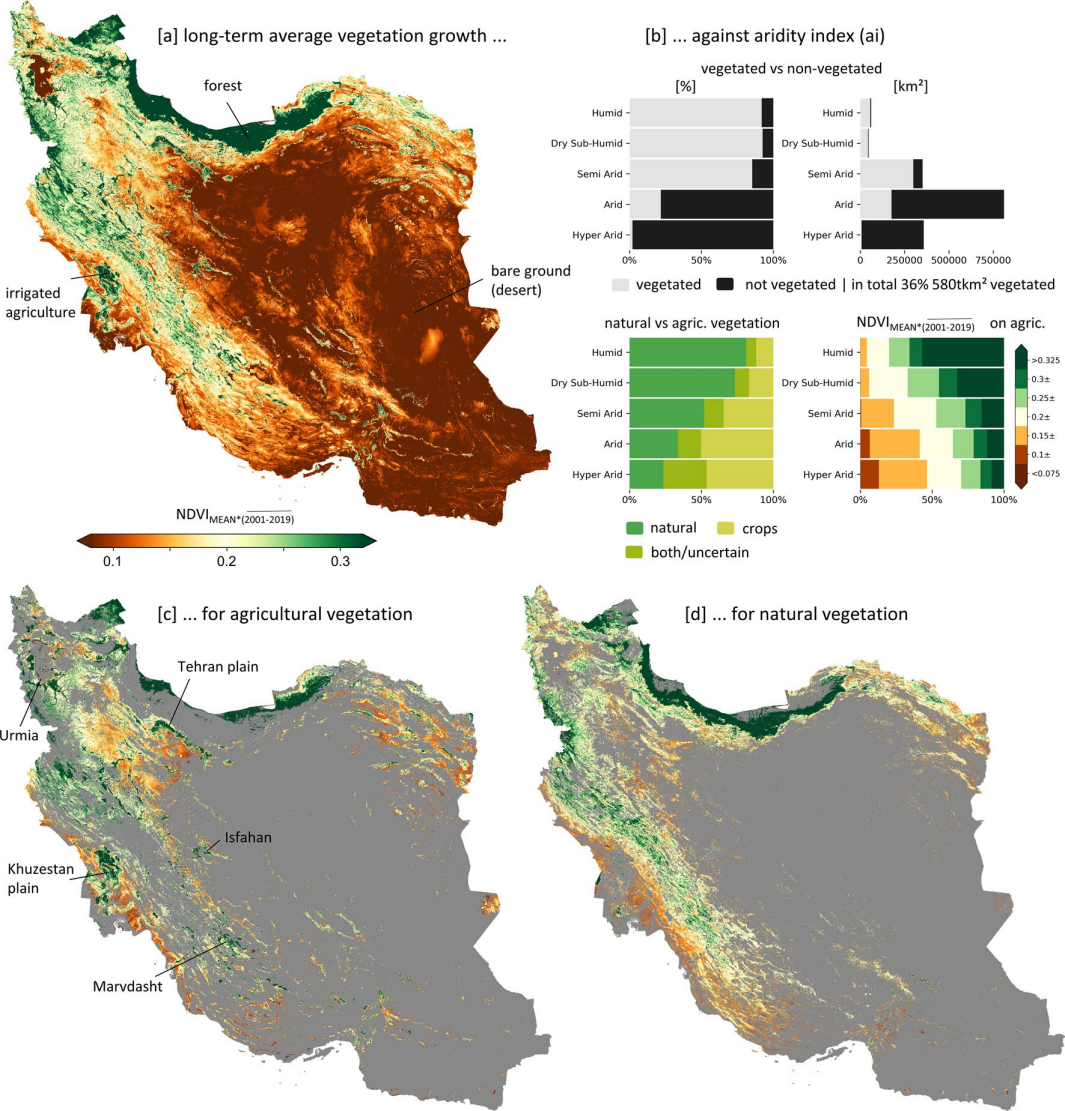
Long-term vegetation growth, i.e. average of the annual vegetation growth ($NDVI_{MEAN}*$) between 2001 and 2019. (**a**): Long-term vegetation growth for entire Iran. (**b**): Vegetation specific statistics related to the aridity index (*ai*). Upper panel: vegetated versus not vegetated areas. Lower left panel: share of natural and agriculture vegetation. Lower right panel: Long-term vegetation growth distribution for agricultural areas. (**c**): Long-term vegetation growth for agricultural vegetation, indicating the locations of some large irrigation networks. (**d**): Long-term vegetation growth for natural vegetation. General Notes: Agricultural areas are identified by the recent global land cover map of Copernicus Global Land Service (Methods). Natural vegetation is defined as being no agricultural land and where the NDVI exceeds 0.25 in at least two water years (Methods). Maps were created using Python 3.9 (https://www.python.org/).

Annual vegetation growth ($NDVI_{MEAN}*$) derived from a MODIS NDVI time series is introduced as a measure for green vegetation during snow free periods within a water year (Methods). High values of $NDVI_{MEAN}*$ correspond to high NDVI values during the whole snow free period of a year indicating dense evergreen forest cover or intense agricultural land use of multiple crop cycles or orchard cultivation. Medium and lower $NDVI_{MEAN}*$ correspond to short-term dense vegetation (e.g. single crop cycles, short vegetation periods) or longer-term vegetation cover with lower NDVI values due to e.g. sparse vegetation or agricultural fields smaller than the 250 m spatial resolution of MODIS. The long-term average of annual $NDVI_{MEAN}*$ values between 2001 and 2019 (Fig. 4) gives further insights into the inter-annual conditions of intra-annual vegetation growth. High long-term averages correspond to permanently high $NDVI_{MEAN}*$ values (e.g. agricultural usage of multiple cropping cycles every year or dense forest cover) and lower values to less cropping cycles during a year, sparse vegetation, small field sizes, or infrequent agricultural usage over the whole time period (2001–2019).

Figure 4a shows clear spatial patterns of long-term vegetation growth, with higher values in the wetter northwest, in the north between the Caspian See and the Alborz Mountains, along the Zagros Mountains, and in agricultural areas distributed all over Iran, e.g. the large irrigation networks in the Khuzestan plain, in the surrounding of Urmia Lake, and in the vicinity of Tehran (Fig. 4b). Very low values can be found in the dry central part of Iran with arid and hyper-arid conditions, representing non-vegetated areas (Fig. 4a). In total, a third of Iran was at least temporarily vegetated during the period from 2001 to 2019 (Fig. 4b). Moreover, clear relationships can be observed between long-term vegetation growth and long-term aridity index (Fig. S5). Under more arid conditions, the proportion of non-vegetated areas increases and the vegetated areas are more and more the result of agricultural usage (Fig. 4b). In general, agricultural areas show higher long-term annual vegetation growth values under more humid conditions, suggesting several cropping cycles within a year for most of the years between 2001 and 2019. However, in Iran, such high values can also be found under semi-arid, arid, and even hyper-arid conditions, where net water deficits would naturally prevent such intense vegetation growth and thus indicate prevailing irrigated agriculture for these areas.

#### Vegetation growth trends

**Figure 5:**
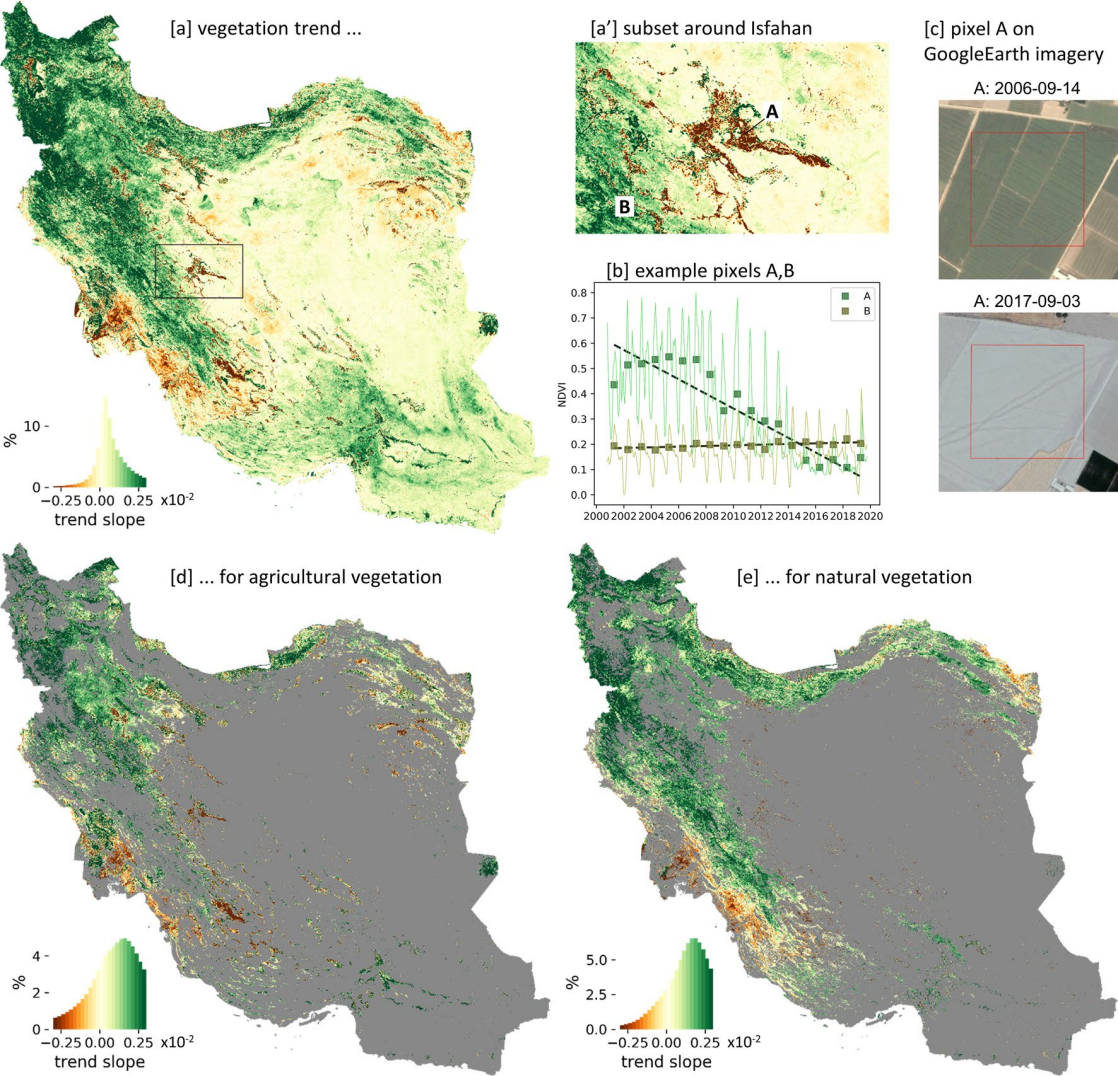
Vegetation growth trends of Iran. (**a**): Vegetation growth trend (trend slope of annual vegetation growth $NDVI_{MEAN}*$ time series between 2001 and 2019) for Iran with subset around Isfahan (**a’**). (**b**): At two example pixels A and B the pre-processed MODIS NDVI time series (line plot), the annual vegetation growth $NDVI_{MEAN}*$ (rectangles), and the trend (dashed line) are given. (**c**): The footprint of the MODIS example pixel A of 250 m $\times$ 250 m is overlaid in red on historical high resolution Google Earth imagery, showing the decrease in agricultural usage. (**d**): Vegetation growth trends for agricultural areas. (**e**): Vegetation growth trends for naturally vegetated areas. Maps were created using Python 3.9 (https://www.python.org/).

The vegetation trends for the period between 2001 and 2019 (Fig. 5), i.e. linear trend slope derived from the annual vegetation growth ($NDVI_{MEAN}*$) time series, show that a vegetation growth decrease is prevalent along the Persian Gulf southeast of Ahvaz and in agricultural areas distributed all over Iran (Fig. 5a’, d). The former corresponds largely with the observed land cover changes from sparse vegetation to bare ground (Fig. 3b). The latter corresponds mainly to large irrigated agricultural areas situated in predominantly arid regions of the country. The (hyper-)arid central part of Iran, almost completely covered by non-vegetated areas, shows largely no vegetation growth trends, also proofing that there are no systematic artifacts in the MODIS data forming the basis of the trend analysis. An increasing vegetation growth trend has been observed for large areas in the wetter northwestern part of Iran and along the Zagros Mountains from the northwest to the southeast reaching almost as far as Shiraz/Marvdasht region. This widespread greening could be observed despite the prevailing warm and drier conditions of the last two decades (Fig. 1), implying an at least partly decoupled relationship between meteorological water availability and annual vegetation growth, and thus to some extent an anthropogenic origin of the observed greening. Estimating the vegetation trends without the dry years at the beginning of the analyzed time period and the wet year 2019 at the end (Fig. 1) still results in widespread positive vegetation growth trends (Fig. S15), which further supports the hypothesis of anthropogenically induced greening.

#### Iran-wide vegetation growth dynamics compared to hydrometeorological conditions

**Figure 6:**
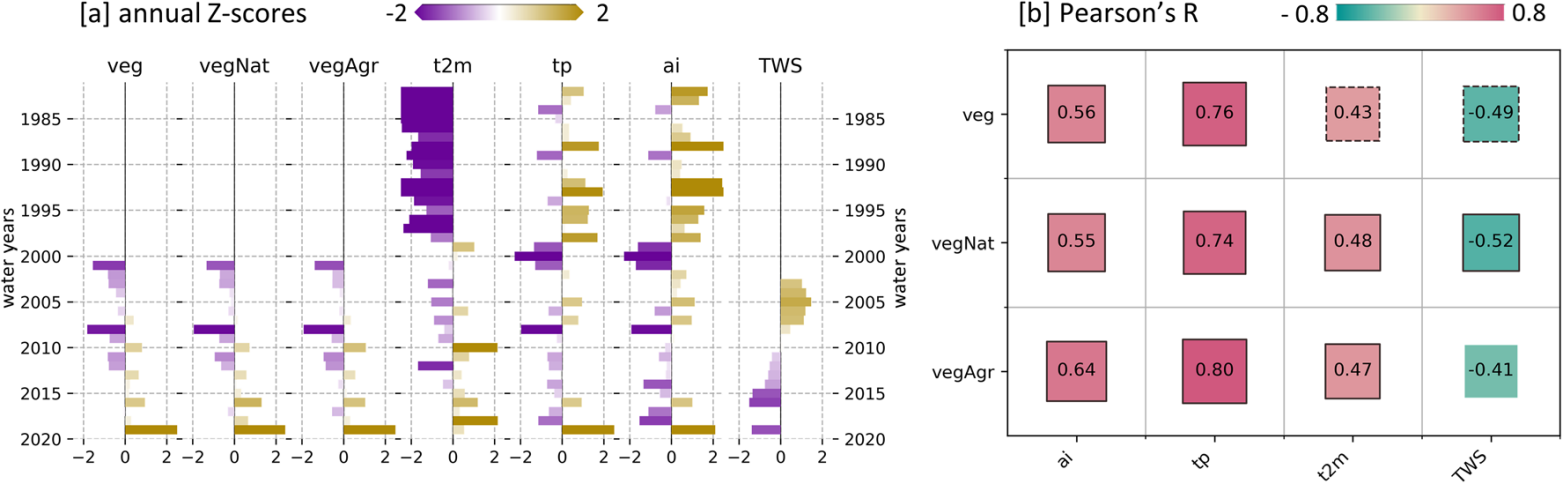
Iran-wide aggregated statistics of vegetation and hydrometeorology. (**a**): Annual Z-scores. Reference period to generate Z-scores applies for all parameters to the common data availability (i.e. period of GRACE *TWS*) (**b**): Pearson’s R values with indication of significance of the correlation (solid boundary: *p* <0.05, dashed boundary: *p* <0.1, no boundary: *p*>=0.1).

Aggregated over entire Iran, the annual vegetation growth dynamics are positively correlated to the water availability (Fig. 6b), revealing that wetter conditions (higher precipitation (*tp*) and aridity index (*ai*)) result in an increased vegetation response (higher $NDVI_{MEAN}*$) and vice versa. This applies very similar for all three analyzed vegetation datasets: $NDVI_{MEAN}*$ on natural vegetation (*vegNat*), on agricultural vegetation (*vegAgr*), and for entire Iran (*veg*) including vegetated and non-vegetated pixels. The highest correlation for all three vegetation datasets can be observed for the precipitation (*tp*) as primary water input (Fig. 6b). Inspecting individual years (Fig. 6a) reveals that the driest years of the analyzed period (2001 and 2008) result in lowest $NDVI_{MEAN}*$ and the wettest year of 2019 in highest $NDVI_{MEAN}*$. In case of the total water storage (*TWS*) the vegetation growth is negatively correlated, showing a vegetation growth increase despite decreasing *TWS*, which suggests unsustainable use of groundwater resources to irrigate vegetation.

#### Natural vegetation growth dynamics compared to meteorological water availability and elevation

**Figure 7:**
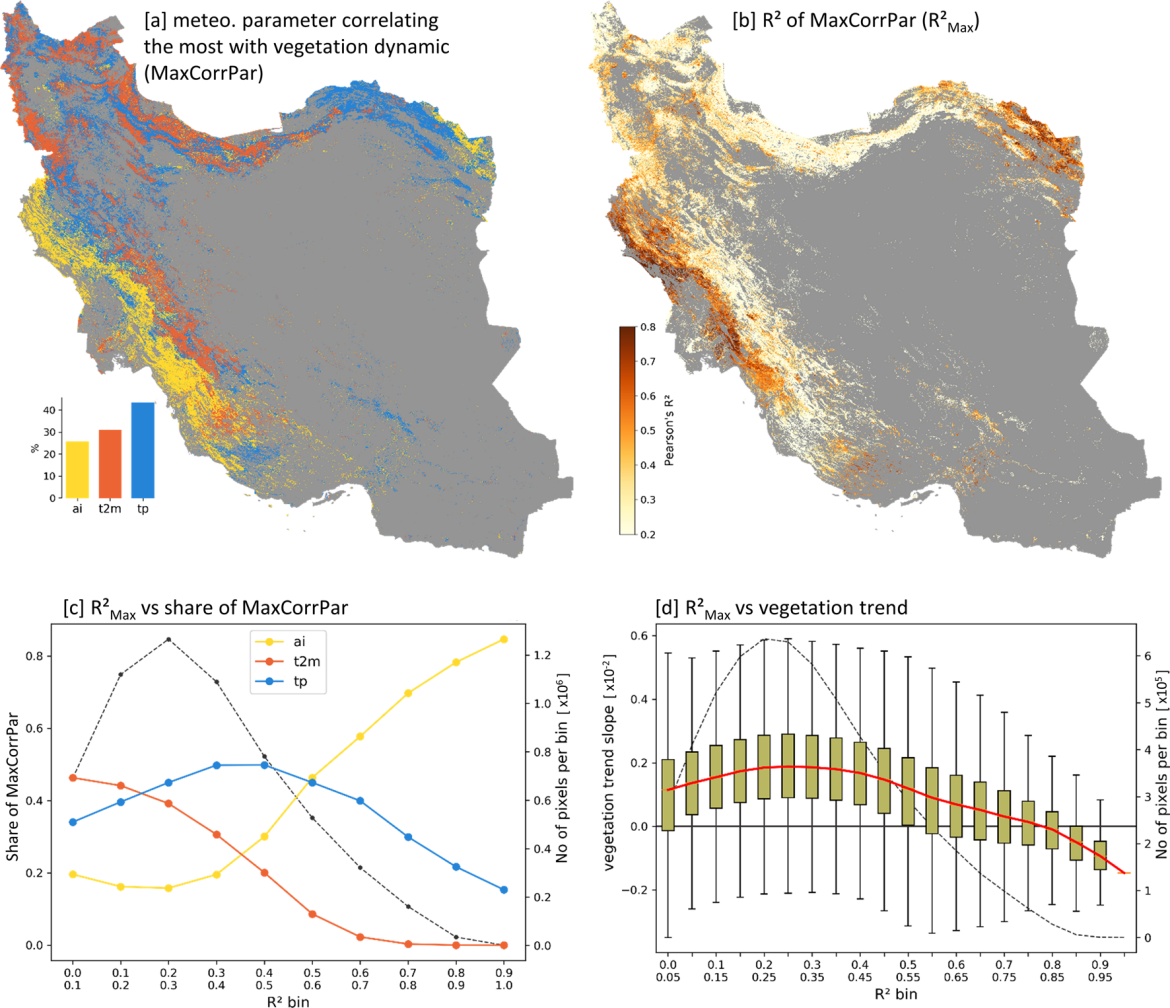
Natural vegetation growth dynamics versus meteorological conditions. Pearson correlation of annual vegetation growth ($NDVI_{MEAN}*$) and annual aggregates of meteorological parameters. (**a**): The map depicts the meteorological parameter that correlates the most (highest $R^2$) with the vegetation growth dynamics at each pixel. (**b**): Pearson’s $R^2$ value of highest correlating meteorological factor ($R^2_{Max}$). Note: individual correlations for each meteorological parameter can be found in Figure S15. (**c**): Proportion of highest correlating meteorological factor per $R^2$ bin (black dotted line: number of pixels per bin). (**d**): Vegetation growth trend (trend slope of annual vegetation growth time series) versus $R^2$ of highest correlating meteorological factor ($R^2_{Max}$). (Per bin a boxplot is shown. Red line: median of the vegetation growth trends per bin. Black dotted line: number of pixels per bin). Maps were created using Python 3.9 (https://www.python.org/).

Natural vegetation is analyzed in relation to meteorological conditions, by correlating the annual vegetation growth ($NDVI_{MEAN}*$) with the annual aggregates of *tp*, *ai*, and *t*2*m* as three parameters mainly controlling the local meteorological water availability. Areas of high correlations between vegetation growth and meteorological parameters can mainly be found in the plains and foothills west of the Zagros Mountains and in the northeast of Iran (Fig. 7b). These areas correlate the most with *ai* (Fig. 7a, c). Since *ai* is a measure of both water input (*tp*) and water loss (potential evaporation), this suggests that such areas are characterized by a rather strong relation to local integrative meteorological water availability. Moreover, it could be observed that higher vegetation-meteorology correlations tend to have decreased vegetation growth trends, with negative slope trends (medians) above an $R^2$ of 0.8 (Fig. 7d). Such areas where vegetation growth is controlled by meteorological water availability are also potentially vulnerable towards future conditions, which are projected to be increasingly warmer and drier. In contrast, there are areas with increasing vegetation growth trends showing low $R^2$ values and thus, seem to be decoupled from meteorological water availability. This suggests either anthropogenic influence or relations to other explanatory variables.

**Figure 8:**
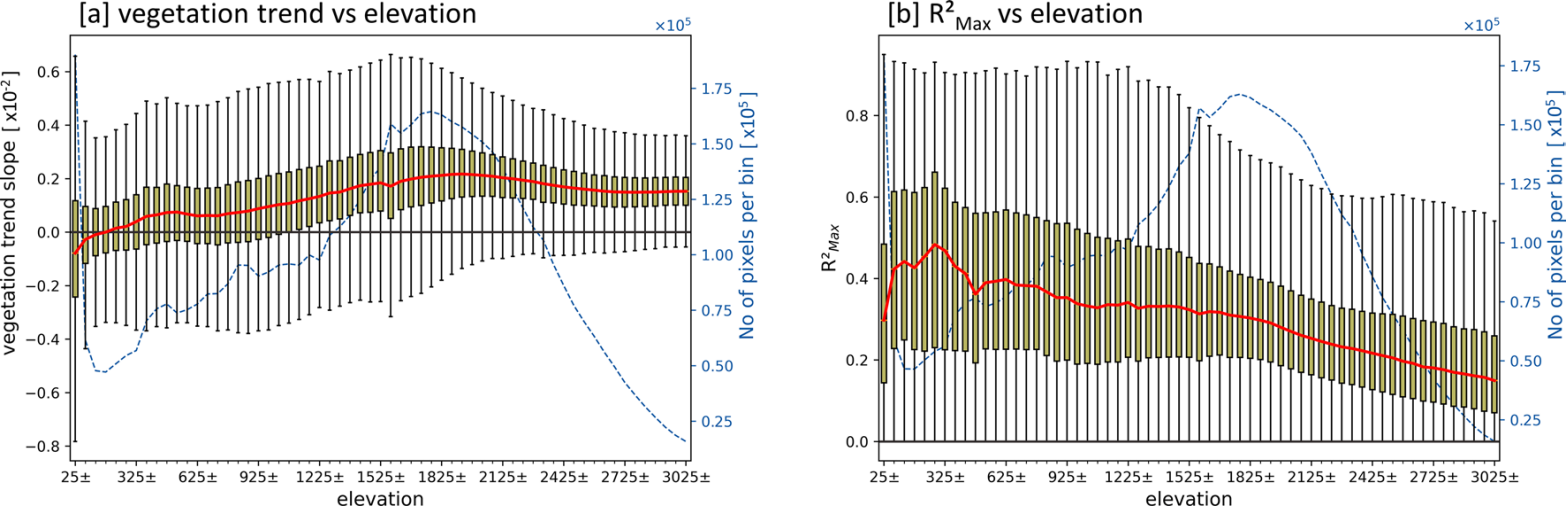
Altitudinal statistics for natural vegetated areas. (**a**): Trend slope of annual vegetation growth ($NDVI_{MEAN}*$) in relation to elevation. (**b**): $R^2$ of the most correlating meteorological parameter ($R^2_{Max}$) in relation to elevation. General note: per elevation bin a boxplot is shown. Red line: median per bin. Black dashed line: number of pixels per bin. A bin represents a 50 m elevation range (e.g. first bin spans from 0 to 50 m altitude). The tick labels of the x-axis represent the middle of a bin.

Relating the vegetation growth trends and the vegetation-meteorology correlations to elevation (Fig. S1), reveals that the lower areas (plains) and foothills of the mountains are characterized by (i) lower vegetation growth trend slopes (Fig. 8a) and (ii) higher $R^2$ values (Fig. 8b) and thus are more closely related to the meteorological water availability. In contrast, higher elevations tend to show (i) higher vegetation growth trend slopes (Fig. 8a) and (ii) lower $R^2$ values (Fig. 8b), revealing that the vegetation growth is more strongly influenced by altitudinal effects.

#### Agriculture vegetation growth dynamics compared to water availability

To assess the temporal development of agricultural areas, we analyze their vegetation growth trends, whereas a positive and a negative vegetation growth trend indicate intensified and reduced agricultural usages, respectively. A third of the vegetation growth trends observed within cropland is statistically significant (*p*-value<0.05), of which approx. 47,500 km$^2$ (83%) are positive and 10,000 km$^2$ (17%) are negative. Iran’s intensively agriculturally used northwestern basins show a strong dominant proportion of intensified agriculture (Fig. S19), with up to 97% of the significant trends being positive (basin IDs 1, 3, 16, 30). In the (hyper-)arid center of Iran the shares of intensified and reduced agricultural areas are more equal, with up to 70% significantly negative trends for basin 24.

**Figure 9:**
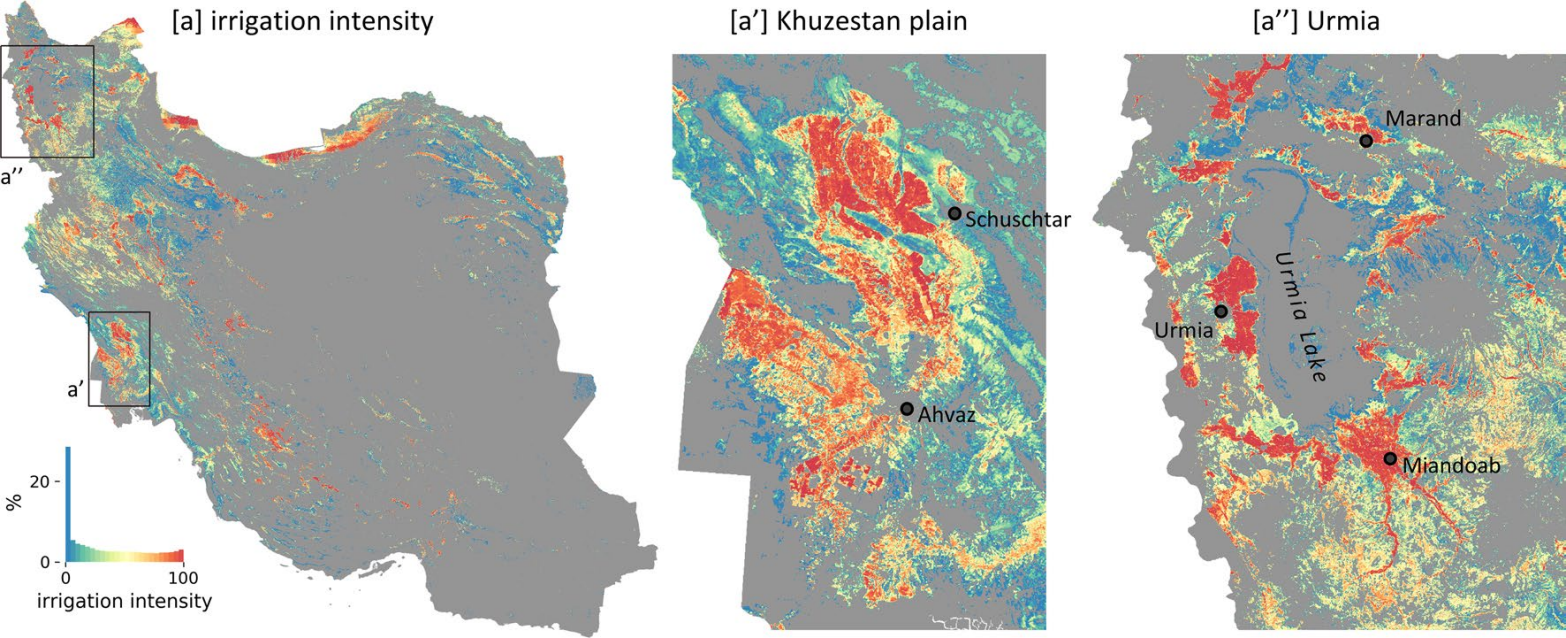
Irrigation intensity as used in Figures 10 and 11. Average of annual irrigation intensities (IrrIntAnn4Q) of the years 2001 and 2003, used to characterize irrigation intensity of agricultural areas already existing at the beginning of the time period of MODIS-based vegetation analysis. (**a**): Map of Iran. Zoom ins: (**a’**): Khuzestan plain (**a”**): Area around Urmia Lake. Note: Derivation of the irrigation intensity is described in methods and in Fig. S20, Fig. S21, and Fig. S22. Maps were created using Python 3.9 (https://www.python.org/).

To evaluate the role of water availability and water usage for the agricultural development, the agricultural areas are characterized by their irrigation intensity (Fig. 9) and aridity (Fig. S5). To characterize irrigation intensity of agricultural areas, we relate the monthly vegetation growth to the meteorological water availability. The monthly irrigation probability (*IrrProb*) is thereby defined as the probability that the observed vegetation growth has required additional non-meteorological water supply in order to grow. *IrrProb* is high, if vegetation is growing under dry conditions and low if growing under more humid conditions. Based on monthly *IrrProb* we calculate annual irrigation intensities (*IrrIntAnn*4*Q*) (Methods). Figure 9 clearly highlights higher irrigation intensity values in regions of larger and smaller irrigation networks across Iran, e.g. in the Khuzestan plain (Fig. 9a’) and around Urmia Lake (Fig. 9a”). It also allows comparison of irrigation intensities over time, such as decreasing or intensified irrigated vegetation growth (Fig. S22). In areas of lower irrigation intensity the vegetation might grow mainly under more humid conditions, which however does not exclude the possibility of irrigation. Moreover, low irrigation intensities might also occur in areas of very sparse vegetation and/or low crop fractions.

**Figure 10:**
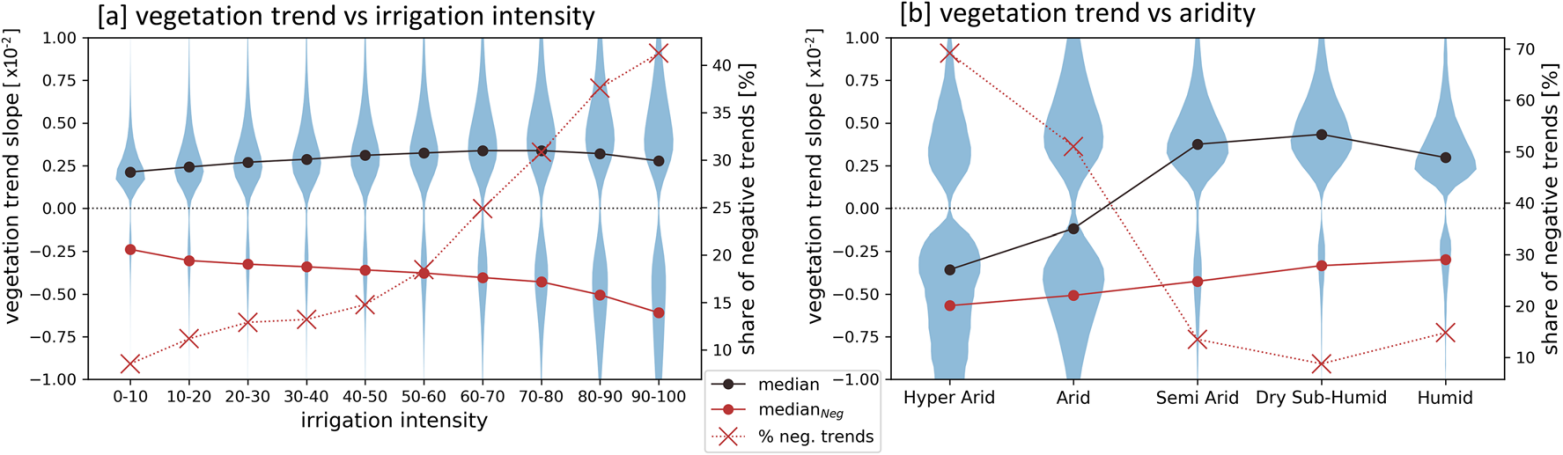
Vegetation growth development on agricultural areas. Trend slopes of annual vegetation growth ($NDVI_{MEAN}*$) are analyzed versus irrigation intensity (**a**) and aridity (**b**). General Notes: Only significant vegetation growth trends (*p* < 0.05) are considered. Agricultural areas which were cultivated after the start of the analyzed period are excluded (Methods). Irrigation intensity of an agricultural pixel is thereby characterized at the beginning between 2001 and 2003 (Fig. 9). The analysis related to aridity focuses on rather highly irrigated areas (irrigation intensity > 50). Black dots: medians of all data points. red dots: medians of negative trends. red crosses: proportion of negative trends.

Analyzing the vegetation growth trends versus irrigation intensities (Fig. 10a), shows that independent from the irrigation intensity most agricultural areas are characterized by a positive vegetation growth trend, indicating an intensified agriculture such as more annual cropping cycles, longer cropping periods, or less periods of no cultivation. However, towards higher irrigation intensities an increasing share of negative vegetation growth trends could be observed implying that high water demand caused by intensive irrigation could have resulted in problems maintaining the agricultural areas. The share of negative vegetation growth trends for intensely irrigated areas (irrigation intensity > 50) are 69% and 51% in hyper-arid and arid areas, respectively (Fig. 10b). In such areas of high aridity, water supply is limited and intense irrigation causes strong unsustainable water usage, resulting in potential longer-term water shortages for agricultural areas, which then are reflected by the observed negative vegetation growth trends.

**Figure 11:**
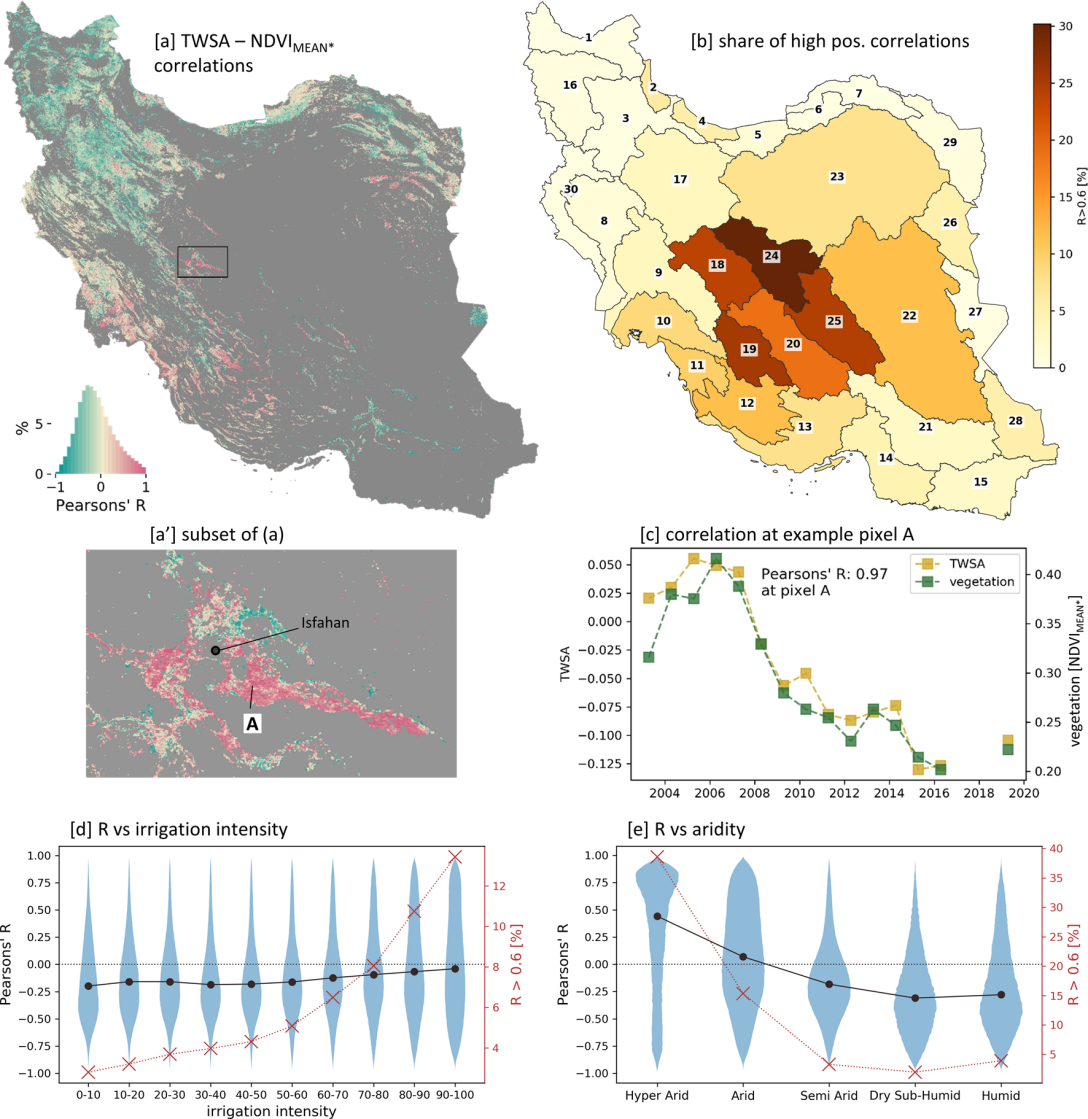
Annual vegetation growth ($NDVI_{MEAN}*$) on agricultural areas compared to annual total water storage anomalies (*TWSA*). (**a**): Map of Pearson correlations between $NDVI_{MEAN}*$ and *TWSA*. (**b**): Share of highly positive correlated (R>0.6) pixels per basin. (**c**): At an example pixel A, the $NDVI_{MEAN}*$ and *TWSA* time series and the associated Pearson’s R is plotted. (**d**): R values versus irrigation intensities. e: R values versus aridity. General Notes for d, e: Agricultural areas which were not existing at the start of the analyzed period are excluded (see methods). Irrigation intensity of an agricultural pixel is thereby characterized at the beginning of the period (average irrigation intensity of 2001–2003, see Fig. 9). The analysis versus aridity focuses on rather highly irrigated areas (irrigation intensity > 50). Black dots: median of all data points. red crosses: proportion of negative trends. Maps were created using Python 3.9 (https://www.python.org/).

To get a deeper insight, where water shortages might have been the controlling factor for a decrease of cropping intensity (i.e. negative trend in annual vegetation growth) we correlate the annual total water storage (*TWS*) of GRACE(-FO) to the annual vegetation growth dynamic ($NDVI_{MEAN}*$) (Fig. 11). Since the *TWS* is decreasing in Iran (Fig. 2), high positive correlations reflect vegetation growth decreases that are probably related to water losses. Areas of high positive correlations can be found especially in the (hyper-)arid central part of Iran (Fig. 11a,b), e.g. around Isfahan (Fig. 11a’). Such high positive relationships get more frequent towards higher irrigation intensities (Fig. 11d) and towards higher aridity (Fig. 11e), suggesting that unsustainable water usage in arid areas results in unsustainable cropping potential/intensity. In contrast, negative correlations, displaying intensified agriculture despite decreasing *TWS*, are dominant in the wetter areas of northwestern Iran (Fig. 11a). Although to a lesser spatial extent, high negative correlations could also be observed in the (hyper-)arid southeastern basins of Iran (Fig. S23), suggesting an intensified cultivation despite its already dry meteorological conditions and a decreasing total water storage.

## Discussion

We have investigated the spatiotemporal dynamics of the last 20+ years of Iran’s land cover, vegetation growth, hydrometeorological quantities, and their interrelations at local and country-wide scales analyzing freely available global climate models, satellite remote sensing data, and its derived products.

### Hydrometeorological developments

Since 1980, Iran has been facing an overall trend towards drier meteorological conditions, whereas an increase in temperature proved to be the main driver rather than a decrease in precipitation (Fig. 1). Thus, our ERA5-Land-based findings go in line with many other studies analyzing long-term meteorological changes in Iran^21,29,30,35,38^. Since the severe drought period from 1999 to 2001, Iran has experienced a 20-year warm and dry period (compared to 1980–2000), with a less pronounced drying trend or even a reverted trend towards wetter conditions. Mehravar et al. (2021)^65^ analyzed integrative drought indices for Iran from 2001 to 2020 and reported trends to wetter conditions even in most basins of Iran.

At the same time, the total water storage (*TWS*) derived by GRACE(-FO) decreased significantly. This strong decrease of *TWS* is not driven by a decrease of precipitation as the primary water input parameter (Fig. 1) and thus is an example of an anthropogenic drought, where water stress is caused or intensified by human activities^66,67^. For Iran, it was recently shown that the water withdrawal dominates climatic and hydrologic processes as influence on surface water availability^68^ and groundwater depletion^69,70^. In this context, Iran is part of the bigger Middle East region showing an overall strong water storage depletion, especially after 2000, whereas Iran is one of the regions with the highest water losses^71^. This unsustainable water withdrawal, especially by groundwater retrieval beyond recharging capacity^9,12,13,22,26^, is known to be driven by the agricultural sector, which is responsible for more than 90% of Iran’s total water consumption^22^.

### Agricultural areas and associated vegetation growth

In Iran an increase of cultivated areas and/or an intensification of existing agricultural areas has been a declared political and economic aim^7,9,11,72^, in order to meet food demands of the increasing Iranian population^8^ under the policy of self-sufficient agriculture, and at the same time to pursue an increase of revenue from the export of agricultural goods^24,72^. We hereby could confirm and spatiotemporally quantify an increase of agricultural areas by 26,771 km$^2$ (9%) from 1992 to 2019 by examining the ESA-CCI land cover datasets (Fig. 3, Table S2). In contrast, the data about annual harvested areas, provided by Iran’s Ministry of Agriculture - Jahad^59^, shows a slight declining trend for a comparable time period between 1990 and 2015. This discrepancy implies that the share of existing farmland being actually harvested is reducing, which could be explained by the fact that agricultural areas are located in unsuitable areas^7^ that do not allow cultivation every year due to unfavorable conditions.

In most agricultural areas positive vegetation growth trends could be observed during 2001 to 2019, implying their intensified cultivation and irrigation. Approximately 47,500 km$^{2}$ of cropland show significant positive vegetation growth trends, representing 83% of the significant agricultural vegetation growth trends and 28% of the whole agricultural area in Iran. A positive vegetation growth trend (i.e. increase in annual vegetation growth $NDVI_{MEAN}*$) can thereby be associated with more annual cropping cycles, longer cropping periods, less periods of no cultivation, or a change towards crop types characterized by higher NDVI response. In accordance, Iran’s Ministry of Agriculture - Jahad reported a stable increase of total crop yield between 1990 and 2015^59^. Such agricultural intensification got particularly evident in the widely cultivated wetter northwestern basins of Iran under mainly semi-arid conditions, where the positive share of significant agricultural vegetation growth trends exceeds 95% per basin (Fig. S19). Besides these wetter and thus more suitable areas for agricultural usage, positive vegetation growth trends are also evident in the center and southeast of Iran under (hyper-)arid conditions, where limits in natural surface water availability and high evapotranspiration rates require intense irrigation for vegetation growth. The long-term average of the annual vegetation growth (Fig. 4) also reveals that with increasing aridity vegetation growth is more and more confined to agricultural usage, with a more than 80% share under hyper-arid conditions. Although, the long-term vegetation growth values generally decrease towards higher aridity, high values, representing long intra- and inter-annual vegetation periods, are also present under such unfavorable arid and hyper-arid conditions. Overall, our study reveals an agricultural expansion and intensification despite long-term decreasing meteorological water availability and a cultivation of (hyper) arid land despite its natural unsuitability for vegetation growth.

Besides the main tendency towards intensified agriculture, a degrading agricultural usage (i.e. cropland with negative vegetation growth trend) could also be observed. In total, 17% (10,000 km$^{2}$) of the significant agricultural vegetation growth trends are negative, representing 6% of all agricultural areas in Iran. The analysis of the vegetation growth trends versus aridity and irrigation intensity (Fig. 10) has revealed an increasing share of negative agricultural vegetation growth trends towards more arid conditions and higher irrigation intensities. The share of significant negative vegetation growth trends on intensively irrigated areas reached approx. 50% and 70% under arid and hyper-arid conditions, respectively. This suggests that in such dry areas unsustainable water use has reached a level of unsustainable cultivation, eventually resulting in reduced agricultural intensity or even uncultivated abandoned fields. Examples where unsustainable water usage led to temporary or permanent abandoned agriculture are e.g. the region around Isfahan^43,73–75^ (Fig. 5) and around Marvdasht and Shiraz^76,77^ (Fig. S22).

The reasons that a former irrigated area cannot be cultivated anymore can be manifold. It is often driven by the reduced water availability and the associated rising efforts to meet the water demand, e.g. accessing deeper ground water horizons or implementing water transfer projects. In the central basins of Iran (Fig. 11) up to 30% of the agricultural area is highly positively correlated with decreasing total water storage (*TWS*), supporting the relation of negative agricultural vegetation growth trends and reduced water availability, mainly due to reduced groundwater storage. However, there are more processes related to irrigation and reduced water availability that might lead to unusable agricultural land, such as degradation of soils (e.g. salinization)^55^, subsidence^47,78^, desertification^79,80^, and sink hole formation because of lowered groundwater tables^81,82^. Until recently, the share of degrading crop areas in the wetter northwest of Iran is rather low, however it might increase in the future if water will continue to be used as unsustainably as in the last decades. The strongly declining water volume of Urmia Lake is one prominent consequence of the intense agricultural water consumption under current climatic conditions^45,83–87^.

### Natural vegetation growth response

This study also analyzed the consequences for natural vegetation under the long-term trend towards drier and warmer meteorological conditions and the ongoing anthropogenic drought. Between 1992 and 2019, formerly sparse vegetation has degraded into bare ground in an area of approx. 40,000 km$^{2}$, dominantly located along the Persian Gulf coast (Fig. 3). For Iran, such large land cover transitions could also be observed by Minaei et al. (2018)^88^, although they were mainly focused on central Iran by comparing two independently generated land cover data sets of GlobeLand30 in 2000 and 2010. In contrast, the ESA-CCI-LC data used in this study has been especially designed for land cover change analysis by relating the annual data classifications to each other and thus, obtaining a data product of high temporal consistency^89^. In addition, we partly validated our land cover data by field visits, visual inspections, and cooperation with local partners, and thus consider our findings more reliable. It needs to be noted, that global land cover data are bound to uncertainties, especially in sparsely vegetated areas^90^, which we cannot sufficiently quantify in this study. However, the identified degrading regions were found to largely correspond to the MODIS-derived negative vegetation growth trends in naturally vegetated areas, confirming the classified land cover transitions towards bare ground.

The analysis of annual natural vegetation growth and meteorological conditions showed stronger correlation if (i) the regression slope of the vegetation growth trends are lower (Fig. 7d), (ii) the aridity index, as an integrative measure of precipitation and temperature-driven evaporation, plays a dominant role (Fig. 7c), and (iii) the areas are located in lower elevations (Fig. 8b). These areas characterized by such strong vegetation-meteorology correlations are also expected to be vulnerable to future climatic changes, which are predicted towards future drying and warming^26,32,33,40–42^, and thus put further pressure on these areas. In contrast, the lower vegetation-meteorology correlations are located at higher elevations (Fig. 8b) and are also characterized by higher vegetation growth trend slopes (Fig. 7d). Thus, the widely observed natural vegetation greening trends (Fig. 5e) are somehow decoupled from the trends in meteorological water availability. This goes in line with the widely reported global trends towards vegetation greening^84–86^, which are accepted as evidence of anthropogenic climate change^91^. Besides this overall long-term greening, Pan et al. (2018)^92^ reported an increasing share of browning trends in the more recent times and argues that greening trends will probably be more and more reverted in a warmer future. At global scale, the main drivers of these greening developments are found to be CO$_{2}$ fertilization, nitrogen deposition, climate change, and land use changes^91,93–95^.

However, especially the widely positive vegetation growth trends in the Zagros Mountains, still seem to be in contrast to the widely reported dieback of the Zagros Oak tree forests^96–100^. Besides a potential role of CO$_{2}$ fertilization, land use changes might have played the biggest role. Since part of the trees have been substituted by agriculture, horticulture, and managed pastures^99,101,102^, the NDVI-based vegetation growth trend of the 250 m MODIS pixels could have increased even if the forest cover got sparser. In this study, the influence of agricultural areas on vegetation growth might be underestimated in the Zagros Mountains, because the dominantly small fields in this area (average sizes of Iran’s irrigated and rainfed farms are 2.9ha and 6.9ha, respectively^11^) could have been missed by the crop fraction layer of 100 m pixel size used in this study to distinguish between cultivated and natural vegetation. Sadeghi et al. (2017)^102^ attributes low classification accuracies of agricultural areas in parts of the Zagros Mountains also to insufficient spatial resolution even in case of 30 m Landsat imagery. Another potential reason for the increasing trend of seasonal vegetation indicators, such as the used $NDVI_{MEAN}*$, could be a prolonged growing season of grassy and bushy vegetation in higher altitudinal ranges caused by the temperature increase of the last decades^103^.

### Iran-wide relations of vegetation and hydrometeorology

At a country-wide scale it could be shown that the annual vegetation growth has a significant positive correlation to annual variations of precipitation and the aridity index (Fig. 6), which applies for both natural and agricultural vegetation. This strong relationship of annual vegetation growth and meteorological water availability also becomes evident in the drastic reduction of vegetation growth in the drought year of 2008 and strong increase of vegetation growth in the wet year of 2019. However, at a local scale this relationship is less clear and as shown in this study, strongly depends on local conditions determined by natural predisposing factors (e.g. lithology, relief (Fig. 8)), local hydrometeorological conditions, and anthropogenic influences (e.g. land management strategies, water withdrawal). By comparing a managed and unmanaged watershed in Iran, Kazemzadeh et al. (2021)^104^ also found strong vegetation cover response to changes in annual natural water availability, but revealed that long term trends were mainly influenced by human activity. For Iran, Dameneh et al. (2021)^105^ also showed that trend estimations for vegetation and climate indices during 2001 and 2015 strongly vary between annual and seasonal aggregates as well as country-wide and pixel-based analysis. Overall, this shows that care needs to be taken in interpreting the influence on vegetation changes, and that factors such as spatial scale, temporal scale (long-term trend vs short-term variability), and observed time period always need to be considered.

## Conclusion

This study enabled detailed insights in spatiotemporal dynamics of water availability and vegetation growth in Iran assessed between 2001 and 2019 for natural and agricultural land on local and country-wide scales. For this purpose we analyzed global data sets on meteorological water availability, total water storage, land cover changes, and vegetation growth dynamics. In conclusion, the study’s main findings are:

- Substantial decline of total water storage is not represented by a decline of meteorological water input (precipitation), suggesting an unsustainable use of groundwater as driver and thus an anthropogenically induced water scarcity in Iran.
- Confirmation and spatiotemporal quantification of agricultural expansion and intensification, playing a key role towards increasing water scarcity due to large and unsustainable consumption of surface water and ground water for irrigation purposes.
- Loss of agricultural areas or reduction of cultivation intensity, as a consequence of water scarcity, especially in former intensely irrigated croplands in (hyper-)arid regions of Iran.
- Widespread degradation of natural sparse vegetation to barren land in the plains west of the Zagros Mountains and at the same time
- Widespread greening of natural biomes especially in higher altitudinal zones following the general global trend towards a greening vegetation.

Such detailed insights in agricultural and natural vegetation developments as well as their interrelation with hydrometeorological factors are an important foundation to support water and ecosystem management plans in Iran^11,19^ to adapt towards a more sustainable future.

## Data and methods

### Hydrometeorology

#### Meteorological data from ERA5-Land

The meteorological data in this study is obtained from the European Center for Medium Range Weather Forecasts^106^. In particular, we are using the land-surface offline re-run of ECMWFs latest atmospheric reanalysis, namely ERA5-Land^107^. ERA5-Land uses atmospheric forcing from the ERA5 reanalysis to consistently estimate hourly land surface variables at an enhanced spatial resolution of 0.1$^{\circ }$. While no observations are directly assimilated during the production of ERA5-Land, they indirectly influence the simulation through the atmospheric forcing of ERA5^106^. Furthermore, air temperature, humidity and pressure are corrected to account for the altitude differences between ERA5 and ERA5-Land grids^107^. Since its release, ERA5-Land has been extensively compared to similar datasets or in situ data^108–110^ while other studies used ERA5-Land as hydrometeorological reference data for bias-correcting seasonal forecasts^111^, as driving data for modeling photovoltaic power^112^, or for deriving agricultural drought indicators^113,114^. Within a similar context, Zandler et al. (2020)^115^ compared the performance of ERA5-Land for assessing NDVI anomalies across peripheral conservation areas of Central Asia and concluded that such reanalysis-based datasets outperform gauge- or satellite-based products and their combinations as they are highly variable and may not be applicable in the analyzed regions. This is somehow expectable as ERA5-Land provides a wide set of consistent land-surface parameters from a single model system while combining parameters from different datasets can introduce further inconsistencies and biases. For this study, we have hence obtained hourly ERA5-Land data^116^ for total precipitation (*tp*), air temperature at 2m (*t*2*m*) and potential evapotranspiration (*pev*). From this hourly data, we then calculate annual averages for the water year in Iran, which covers the period from October to September. The aridity index (*ai*) is calculated as the ratio of annual aggregates of *tp*/*pev*^117^. While the analyses in this study are based on ERA5-Land only, we also evaluate long-term trends from other precipitation datasets in order to verify our findings. In particular, we apply the widely used global precipitation data from the Global Precipitation Climatology Centre (GPCC full data monthly version 2020^118^) as well as the Multi-Source Weighted-Ensemble Precipitation dataset (MSWEP^119^), which merges gauge, satellite, and reanalysis data for deriving a global precipitation dataset with high spatial and temporal resolution. Comparison of the trends of the different precipitation products shows a generally good spatiotemporal fit (Fig. S6) and thus supports the reliability of our meteorological analysis performed in this study.

#### Total water storage from GRACE and GRACE-FO

The Gravity Recovery And Climate Experiment (GRACE) satellite mission was launched in March 2002 within the collaboration between the National Aeronautics and Space Administration (NASA) in the US and the German Aerospace Center (DLR)^120^. The mission has provided unprecedented observations of the time variable Earth’s gravity field by tracking changes in the inter-satellite distance between twin satellites via microwave measurements^121^. The GRACE mission has observed the Earth for more than 15 years and ended in June 2017. Its legacy is continued by the successor GRACE Follow-On (GRACE-FO) mission starting in June 2018. The GRACE and GRACE-FO missions provide a unique opportunity to assess and quantify anthropogenic and climatic impacts on the water system^122–124^. In this study, we obtain the Total Water Storage Anomaly (*TWSA*) from GRACE and GRACE-FO level 2 products (unconstrained fully normalized spherical harmonic coefficients) from the ITSG-Grace2018 solution^125,126^ and apply the following corrections known as post-processing steps^127^:

- $C_{20}$ and $C_{30}$ are replaced by the estimation from Satellite Laser Ranging (SLR)^128,129^
- Degree 1 coefficients are added to the solutions^130^
- Correcting spherical coefficients to ellipsoidal coefficients following the approach proposed by^131^
- Removing the long-term mean (2004–2010) as a representative of the static field to calculate anomalies
- Removing the remaining primary and secondary tidal aliasing errors using a least-squares spectral analysis following^132^
- Applying Gaussian filter with radius 400 km^133^ and the de-striping proposed by^134^
- Correcting leakage by applying the data-driven approach developed by^135^
- Removing the GIA using the ICE6G-D model provided by^136^

The aggregated *TWSA* time series are calculated for the entire Iran and its 30 major river basins at monthly time steps. The GRACE mission does not provide solutions for 24 months, leading to a data outage in the time series mainly after 2011 that are filled using the Spline interpolation method^137^. Moreover, an 11-month gap (July 2017–May 2018) exists between the GRACE and GRACE-FO missions. Several studies have already proposed methods to bridge the gravity data gap e.g. using Swarm orbital measurements^138–141^, Singular Spectrum Analysis (SSA) by^142^, and machine learning^143,144^. In this study, we did not fill the mission gap because none of the studies have shown convincing superiority, and it is not possible to examine the uncertainties in the absence of independent other data sources. Therefore, two water years (2017 and 2018) are excluded from our analysis. To obtain the annual Total Water Storage Anomaly (*TWSA*), monthly data is averaged for a water year (Oct–Sept). It should be also noted that the boundaries of the basins are the same as already been employed in^28^.

### Land cover

The analysis of land cover developments uses the global ESA-CCI land cover datasets (ESA-CCI-LC v.207 & v2.1)^145^, which comprise annual land cover data sets from 1992 to 2019 with a pixel size of 300 m. It distinguishes 22 classes, which have been defined using the United Nations Food and Agriculture Organization’s (UN FAO) Land Cover Classification System. It is a well-established land cover data product enabling analysis of large areas up to global scale land cover changes^89^ related to different applications, such as the impact of land cover changes to different landscapes^146^, plant functional types^147^, and the consequences of global urban expansion to cropland productivity^148^. To create this dataset, the entire Medium Resolution Imaging Spectrometer (MERIS) Full and Reduced Resolution archive from 2003 to 2012 was first classified into a unique 10-years baseline land cover data set. Changes from this baseline are detected from Advanced Very-High-Resolution Radiometer (1992–1999), SPOT-Vegetation (1998–2012), and PROBA-Vegetation and Sentinel-3 OLCI data (since 2013). Changes need to be present at least for two years to be identified as change. Thereby, longer lasting abrupt changes are better identified than gradual changes. In 1994 AVHRR data is missing and thus changes around that year could not reliably identified. We validated the ESA-CCI-LC by field work in Khuzestan in 2019, local expertize, and high resolution optical satellite data, and decided to merge the original 22 classes to 7 compound classes in order to eliminate misclassifications between some of the 22 original classes. Frequent misclassifications could be found e.g. between irrigated and rainfed agriculture, and between different sparsely vegetated land cover classes. Table S1 gives an overview on the reclassification scheme resulting in the used 7 classes: crops, trees, shrubs/sparse vegetation/grassland, inundated, urban, bare, and water. However, even after reclassification some limitations exist, which have to be taken into account when analyzing land cover from a global product in a regional context. The forest in the Zagros Mountains e.g., with sparse trees and lower canopy cover of trees, is not mapped as forest, since it does not meet the applied forest definition of a closed canopy of trees.

### Vegetation

#### Data and pre-processing

Analysis of vegetation dynamics is based on the MOderate Resolution Imaging Spectroradiometer (MODIS)^149^. It provides consistent nearly daily acquisitions of 250 m pixel size since 2000 and thus the best compromise of long-term consistency and duration, temporal repetition rate, and spatial resolution for the purpose of analyzing dynamics of vegetation patterns at country-wide scale. Landsat data, with spatial resolution of 30 m and data coverage exceeding the last two decades seems to be the better choice for analyzing local patterns over longer periods of time. However, the 16 days repeat cycle in combination with cloud cover and in earlier times’ occasionally missing data acquisitions, lead to larger temporal gaps of up to several months in the time series introducing potential artifacts in the analysis of vegetation dynamics. Hence, this study is based on the 16 days composites of the daily NDVI (Normalized Difference Vegetation Index) measurements provided by the MOD13Q1 V6 product^150,151^. To reconstruct high-quality NDVI time series from the original MODIS product, the local kernel-based time series smoothing algorithm from Chen et al. (2004)^152^ is applied. Compared to global smoothing algorithms that fit predefined functions to the time series (e.g. asymmetric Gaussian function fitting, and double logistic function fitting) to emulate seasonal phenology, local smoothing algorithms are better suited to preserve intra-seasonal variations and variable time series patterns^153–155^, which both are common for agricultural areas representing the main focus of this study. As a drawback, local smoothing algorithms are more susceptible to outliers^156^. Thus, we combine the local smoothing algorithm of Chen et al.^152^ with a preceding outlier removal technique to create high quality NDVI time series. The workflow comprises the following steps: (I) Pixels representing clear outliers are masked based on the Detailed QA layer of the MODIS product using the parameter setting: $VIQuality>2\ \& \ Possible \ snow/ice == 1$. This masking approach allows to exclude outliers and at the same time to maintain as many as possible pixels in the time series for further analysis. (II) The masked pixels are reconstructed by applying a linear interpolation using the closest in time non-masked acquisitions in the NDVI time series. (III) The resulting reconstructed NDVI time series represents the input for the local time series smoothing algorithm of Chen et al. (2004)^152^, which is based on the Savitzky-Golay filter to iteratively make the data approaching the upper NDVI envelope, assuming that noise such as clouds or poor atmospheric conditions decrease NDVI values.

#### Derivation of annual vegetation growth

The estimation of vegetation dynamics and trends is based on annual aggregated time series rather than the complete time series. In Forkel et al. (2013)^157^ such annual aggregates (e.g. maximum, or mean NDVI value per year) have proven to allow a more robust trend estimate compared to the decomposition of the full time series. This study introduces the annual aggregate $NDVI_{MEAN}*$, which represents the average NDVI during the snow free period of a water year (Oct–Sept). The snow period is excluded to account for artifacts in the NDVI time series introduced by large negative NDVI values resulting from snow cover. The duration of the snow period is derived from the MODIS snow cover product (MOD10A1 V6)^158^, and is defined as the highest number of annual snow days from all water years between 2001 and 2019 (*MaxSnowDays*)(Fig. S13). A snow day is thereby determined by the parameter $NDSI\_Snow\_Cover$ of the MODIS product ($NDSI\_Snow\_Cover > 20$). The $NDVI_{MEAN}*$ of a pixel is defined as the average of the highest *X* NDVI values of the available 23 acquisitions during a water year (Oct-Sept), whereas $X =23-MaxSnowDays/23-1$. Thus, *X* as the number of MODIS NDVI values that are included in the annual aggregate $NDVI_{MEAN}*$ varies in space (per pixel) but not in time (same for each year). The inter-annual fixation of *X* allows for a robust determination of vegetation growth dynamics under annually differing snow conditions.

The integrative nature of the $NDVI_{MEAN}*$ (i.e. averaging over the full vegetation period) reflects also changes in the length of vegetation season or the number of cropping cycles, which is not possible by using a single parameter, such as the maximum of the season. The restriction to the snow free period allows to exclude interpolation artifacts introduced by long snow periods and supports the robust application of this parameter to the entire Iran.

The long-term vegetation growth (Fig. 4) is defined as the average of annual vegetation growth values $NDVI_{MEAN}*$ between 2001 and 2019. This parameter is introduced to analyze the spatial patterns of vegetation growth and its relation to aridity conditions.

#### Differentiation between agricultural and natural vegetation

A pixel is defined as vegetated if the annual NDVI maximum ($NDVI_{MAX}$) of that pixel exceeds 0.25 in at least two years between 2001 and 2019. These vegetated pixels are further differentiated between agricultural and natural vegetation based on the fractional cropland layer of the land cover product of the Copernicus Global Land Service (CGLS-LC100)^159,160^. The fractional cropland layer has a spatial resolution of 100 m and provides fractions of that pixel being cropland^161^. In contrast to discrete classes, these fractions allow a smooth spatial resampling to the MODIS resolution using an average interpolation approach. The land cover product is available annually since 2015. This study bases the decision of a pixel being cropland on the maximum cropland fraction between 2015 and 2019 (Fig. S14), ensuring also the integration of infrequent cultivation. A MODIS pixel is considered as cropland if the resampled aggregated cropland fraction (*CropFrac*) exceeds 20%. This rather low threshold is selected to account also for the small field sizes frequently existing in Iran^11^. In contrast, a MODIS pixel is defined as natural vegetation if *CropFrac* falls below 40%. Hence, the definitions of agricultural and natural vegetation slightly overlap, and pixels characterized by a *CropFrac* between 20% and 40% are included in both definitions. This overlap is chosen to account for mixed pixels and for uncertainties in the CGLS-LC100 product generation.

#### Vegetation growth trend estimation

Based on the time series of annual vegetation growth $NDVI_{MEAN}*$ from 2001 to 2019, the vegetation growth trend estimation is performed by a Mann-Kendall trend test^162^ along with the Theil-Sen’s slope estimator^163,164^ to obtain the magnitude of trend. The non-parametric Mann–Kendall trend test is used to test for monotonic trends (consistently increasing or decreasing) within a time series. In its original version the test requires the time series being free of autocorrelation and seasonality. Through annual aggregation, the used time series is free of seasonality. However, the vegetation growth dynamics over time can partially be autocorrelated, e.g. a severe drought event in one year can have effects on water availability and thus also on vegetation development during the next years. Therefore, we apply the Mann–Kendall trend test of Hamed and Rao (1998)^165^, which addresses autocorrelation by a variance correction approach.

#### Iran wide correlation of vegetation growth and hydrometeorological variables

The correlation of vegetation and hydrometeorology (Fig. 6) is based on a Pearson correlation between the time series of the annual vegetation growth $NDVI_{MEAN}*$ and the annual averages of the hydrometeorological parameters (*tp*, *t*2*m*, *ai*, *TWS*). Both, vegetation and hydrometeorological parameters, are averaged over the entire Iran for each year. The vegetation parameter is distinguished in three classes, (i) *veg*: all pixels in Iran, (ii) *vegAgr*: agricultural areas, and (iii) *vegNat*: natural vegetation.

#### Natural vegetation growth versus meteorological water availability

For pixels defined as naturally vegetated a Pearson correlation is performed between the $NDVI_{MEAN}*$ and annual averages of the meteorological parameters (*tp*, *t*2*m*, *ai*), controlling the meteorological water availability. To allow for a pixel-to-pixel based correlation, the pixels of the meteorological parameters of 0.1$^{\circ }$ spatial resolution are resampled to MODIS’ 250 m spatial resolution using nearest neighbor interpolation.

#### Altitudinal analysis

This study uses the MERIT DEM (Multi-Error-Removed Improved-Terrain Digital Elevation Model)^166^. The MERIT DEM is a global DEM at 3 arc second resolution produced by eliminating major error components (absolute bias, stripe noise, speckle noise, and tree height bias) from existing DEMs (NASA SRTM3 DEM, JAXA AW3D DEM, Viewfinder Panoramas DEM). The MERIT DEM is resampled to MODIS resolution using bilinear interpolation.

#### Agricultural vegetation growth versus hydrometeorological water availability

The agricultural areas are analyzed in regard to their vegetation growth trends in order to determine intensified or reduced agricultural usage. For a consistent analysis, the agricultural areas already need to exist at the start of the analyzed vegetation time period (2001–2019). Areas which were turned into cropland afterwards, are excluded from the analysis to avoid misinterpretations of the related vegetation growth trends. Agricultural pixels (for definition see above) are defined as newly cultivated when: $(MedNDVI_{MAX}f3-MedNDVI_{MAX}l10)>0.1\ \& \ MedNDVI_{MAX}f3<0.3$, whereas $MedNDVI_{MAX}f3$ is the median of the annual NDVI maximum of the first 3 years (2001–2003) and $MedNDVI_{MAX}l10$ is the median of the annual NDVI maximum of the last 10 years (2009–2019). Applying these criteria excludes clear transitions of bare land into cropland. It also excludes cropland which has not been cultivated at the beginning of the time period but turned into cultivated cropland later on (Fig. S18). Thus, the definition of early-on existing agricultural areas utilizes both, the recent high resolution CGLS-LC100 product and the MODIS based NDVI change analysis. This combined approach is chosen over a customized cropland identification based on the MODIS NDVI data itself due to expected high cropland classification uncertainties, because of the wide range of existing variable crop types and management processes under Iran’s diverse natural conditions and due to common field sizes smaller than the MODIS pixel size^11^.

The vegetation growth trends of agricultural pixels are further analyzed in regard to their long-term aridity and irrigation intensity (Figs. 10, 11). The long-term aridity is defined by the annual (water year) average of the aridity index between 1982 and 2019 (Fig. S5). The parameter irrigation intensity is originally defined within this study. It represents an aggregated measure of monthly irrigation probabilities (*IrrProb*), which describe the probability that observed vegetation growth of a month has required additional non-meteorological water supply in order to grow. The *IrrProb* of a pixel is defined as: $$VegProb*CropProb*WaterProb$$ (Fig. S20, S21). It is scaled between 0 and 1, whereas 1 is the highest probability being irrigated. It is based on the assumption that vegetation on cropland under dry conditions is irrigated. *VegProb* is the probability of a pixel being vegetated. For *VegProb* the 16 days MODIS NDVI data is resampled to monthly composites using the maximum monthly NDVI. The probability is scaled between 0 and 1 and is defined by a cumulative distribution function of an exponential distribution: $$VegProb=1-exp(-(NDVI-0.2)/0.08,\ 0\ if\ NDVI<0.2$$ (Fig. S20). *CropProb* is the probability of a pixel being cropland. It uses the resampled fractional cropland layer (*CropFrac*) (Fig. S14) and is scaled between 0 and 1: $$CropProb=(CropFrac -20)/(40-20)+1,NaN\ if\, CropFrac<20,\ 1\ if\ CropFrac>40$$ (Fig. S20). *WaterProb* is the probability that crops need to be irrigated to grow. *WaterProb* is based on the meteorological water availability defined by the two month aridity index ($$ai_2$$), calculated over the current and previous month, following the assumption that vegetation growth is largely driven by cumulative water availability. The probability is scaled between 0.2 and 1, allowing a small probability of the need for irrigation also in wetter conditions: $$WaterProb=1-(ai_2-0.2)/(0.65-0.2)*0.8,\ 1\ if\ ai_2<0.2,\ 0.2\ if\ ai_2>0.65$$ (Fig. S20). Annual aggregates are derived from the monthly irrigation probability (*IrrProb*) to define annual irrigation intensities by averaging the upper 4th quantile of the 12 *IrrProb* month values (*IrrIntAnn*4*Q*) (Fig. S21). To analyze the agricultural vegetation growth development in regard to its irrigation intensity (Figs. 10, 11), agricultural pixels are characterized by its initial state of irrigation intensity at the beginning of the MODIS time period (2001–2019). The initial state of the irrigation intensity is thereby defined as the average of annual irrigation intensities (*IrrIntAnn*4*Q*) of the years 2001 and 2003, and is described by the term irrigation intensity. It needs to be noted that a scaling factor of 100 is applied to the irrigation intensity depicted in Figs. 9, 10 and 11.

The correlation between total water storage (*TWS*) from GRACE and vegetation development (Fig. 11) is based on the Pearson correlation between the annual (water year) time series of *TWSA* (annual averages) and the annual vegetation growth $NDVI_{MEAN}*$. The $NDVI_{MEAN}*$ of a MODIS pixel is correlated against the *TWSA* of the basin the pixel is located in.

## Supplementary Information

Supplementary Information.

## Data Availability

The datasets used and/or analysed during the current study available from the corresponding author on reasonable request. All datasets are also publicly available. Meteorological data from ERA5-Land are accessed from the Copernicus Climate Change Service (https://cds.climate.copernicus.eu/cdsapp#!/dataset/reanalysis-era5-land, DOI: 10.24381/cds.e2161ba). GRACE(-FO) data are accessed from the Hydrosat repository (http://hydrosat.gis.uni-stuttgart.de, DOI:10.5194/essd-2021-174). MODIS 16 day vegetation composites (MOD13Q1 V6 product) used for vegetation analysis are accessible at https://lpdaac.usgs.gov/products/mod13q1v006/ (DOI: .5067/MODIS/MOD13Q1.006). MODIS daily snow cover product is accessible at https://nsidc.org/data/MOD10A1/versions/6 (DOI: 10.5067/MODIS/MOD10A1.006). The annual land cover datasets (ESA-CCI-LC v2.07 & v2.1) are accessed from the Copernicus Climate Change Service (https://cds.climate.copernicus.eu/cdsapp#!/dataset/satellite-land-cover). The fractional cropland layer of the land cover product of the Copernicus Global Land Service (CGLS-LC100) is available at https://zenodo.org/record/3939050 (DOI: 10.5281/zenodo.3939050). The MERIT DEM (Multi-Error-Removed Improved-Terrain Digital Elevation Model) is accessible at http://hydro.iis.u-tokyo.ac.jp/~yamadai/MERIT_DEM/.
