# Supplementary material for: Interrelations of vegetation growth and water scarcity in Iran revealed by satellite time series
Source: Sci Rep. 2022 Dec 1;12:20784. doi: 10.1038/s41598-022-24712-6 (PMC9715656; doi:10.1038/s41598-022-24712-6)
Supplement: Supplementary file 1 — Supplementary Information. [file 41598_2022_24712_MOESM1_ESM.pdf]

Interrelations of vegetation growth and water scarcity in Iran revealed by satellite time series

Robert Behling[1,*], Sigrid Roessner[1], Saskia Foerster[1], Peyman Saemian[2], Mohammad J. Tourian[2], Tanja C. Portele[3], and Christof Lorenz[3]

[1]Helmholtz Centre Potsdam GFZ German Research Centre for Geosciences, Remote Sensing and Geoinformatics Section, Potsdam, Germany
[2]University of Stuttgart, Institute of Geodesy, Stuttgart, Germany
[3]Karlsruhe Institute of Technology (KIT), Campus Alpin, Institute of Meteorology and Climate Research - Atmospheric Environmental Research (IMK-IFU), Garmisch-Partenkirchen, Germany
[*]robert.behling@gfz-potsdam.de

The supplement provides figures and tables supporting the results, methods, and discussions of the main manuscript and follows the general structure of the main manuscript.

S.1 General Iran maps
Fig S 1 – Map of Iran.
Fig S 2 – Water basins of Iran.

S.2 Hydrometeorology
Fig S 3 – Annual average precipitation of Iran.
Fig S 4 – Annual average temperature of Iran.
Fig S 5 – Long-term aridity index.
Fig S 6 – Comparison of precipitation products.
Fig S 7 – Significance of meteorological trends.
Fig S 8 – Meteorological trends during GRACE(-FO) period.
Fig S 9 – Significance of meteorological trends during the GRACE(-FO) period.
Fig S 10 – Annual Z-score maps of ERA5-Land total precipitation (tp).
Fig S 11 – Annual Z-score maps of ERA5-Land temperature (t2m).
Fig S 12 – Annual Z-score maps of ERA5-Land aridity index (ai).

S.3 Land cover
Table S 1 – Reclassification scheme of the ESA CCI land cover product.
Table S 2 – Land cover change between 1992 and 2019.

S.4 Annual vegetation growth $NDVI_{MEAN*}$ ancillary data
Fig S 13 – $MaxSnowDays$.

S.5 Differentiation of agricultural and natural vegetation ancillary data
Fig S 14 - Crop fraction per MODIS pixel.

S.6 Trends and annual dynamics of vegetation
Fig S 15 – Vegetation trend between 2005 and 2018.
Fig S 16 – Annual Z-score maps of vegetated pixels.

S.7 Natural vegetation compared to meteorological conditions
Fig S 17 – natural vegetation dynamics against meteorological conditions.

S.8 Agricultural vegetation compared to hydrometeorology
Fig S 18 – newly cultivated areas per basin.
Fig S 19 – Agricultural areas and associated vegetation trends per basin.
Fig S 20 – Visualization of the calculation rules for VegProb, WaterProb, and CropProb.
Fig S 21 – Irrigation probability derivation.
Fig S 22 – Comparison of irrigation intensities from 2001-2003 and 2017-2019.
Fig S 23 – Correlation between annual vegetation growth ($NDVI_{MEAN*}$) and annual total water storage anomalies (TWSA) for agricultural areas.

S.1 General Iran maps

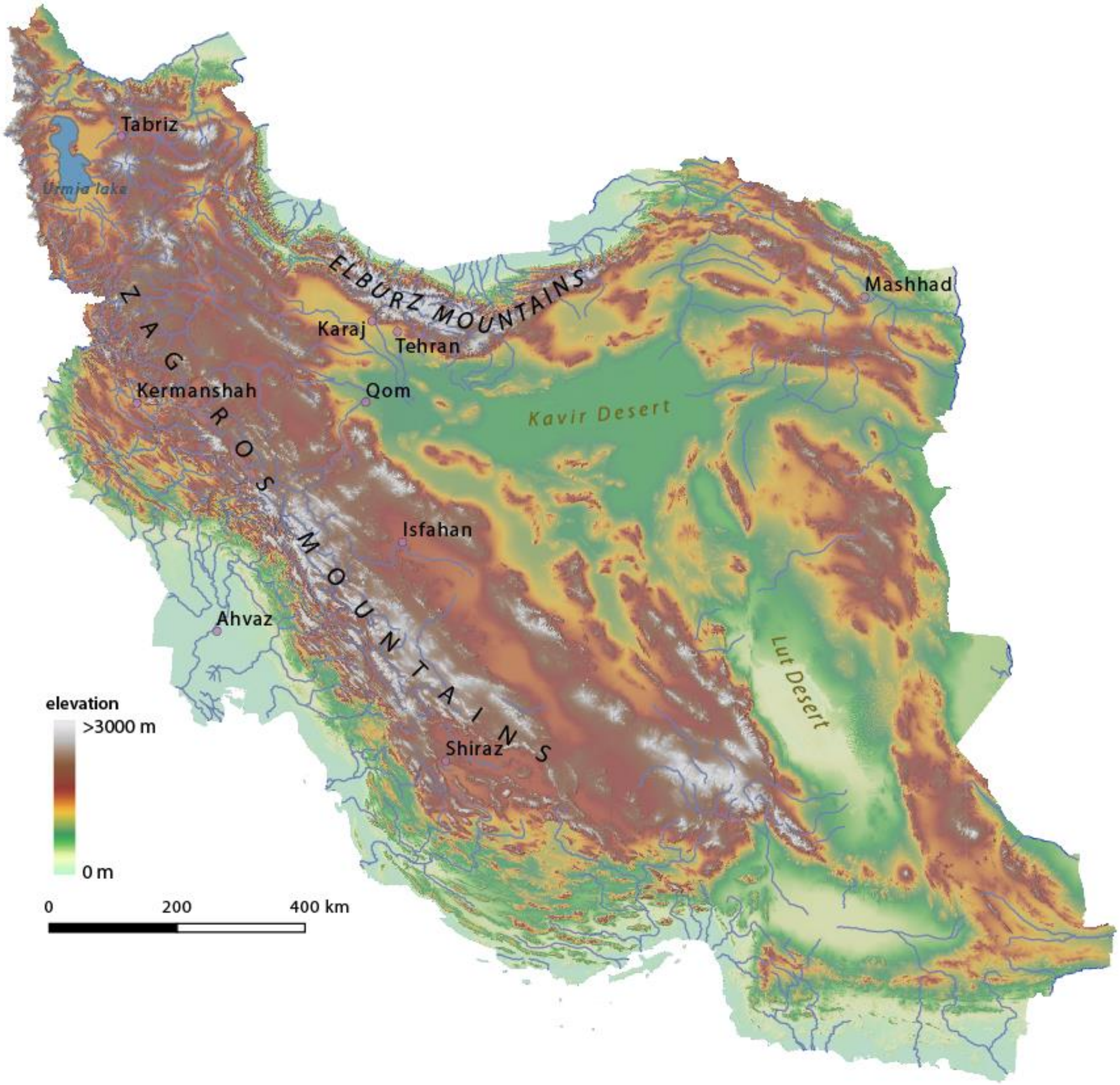

Fig S 1 – Map of Iran indicating topographical features and areas mentioned throughout the manuscript. Map was created using QGIS 3.10 (https://qgis.org/).

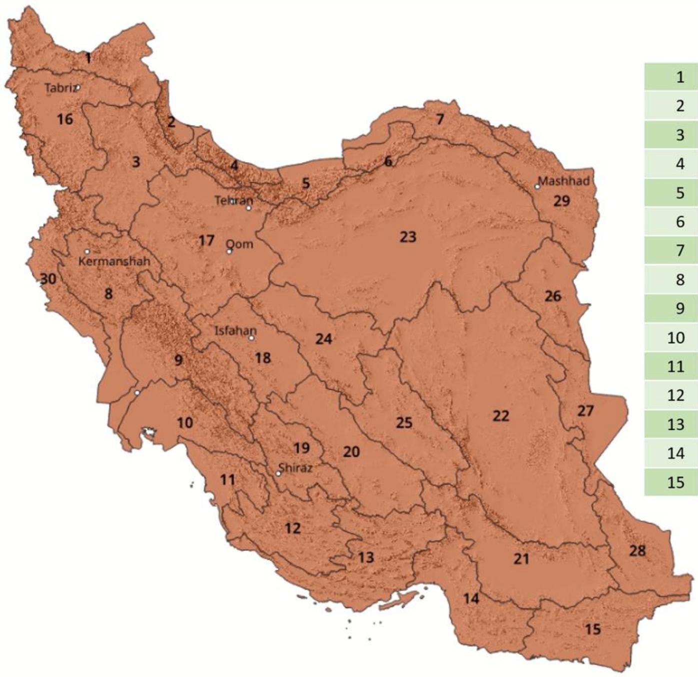

1	Aras river	16	Urumia lake
2	Talesh-Anzali Swamp	17	Namak lake
3	Sepidrood river	18	Gavkhuni
4	Sepidrood river va haraz river	19	Tashk, Bakhtegan, Maharlu
5	Haraz river to Qaresou river	20	Abarghoo- Sirjan
6	Qaresou river and Gorgan rood	21	Hamoon, Jazmoorian
7	Atrak river	22	Kavir Lut
8	Karkhe river	23	Kavir markazi
9	Karun-Bozorg river	24	Siahkoh Kavir
10	Jarahi river and Zohreh River	25	Kavir- Daranjir
11	Haleh-Rood river	26	Khaf-salina
12	Mand river	27	Hamoon-Hirmand
13	Kol river- Mehran river	28	Hamoon-Meshkil
14	Bandar abbas-sedich	29	Karakum
15	Southern Baluchestan	30	Boundary rivers on west

Fig S 2 – Water basins of Iran. Map was created using QGIS 3.10 (https://qgis.org/).

S.2 Hydrometeorology

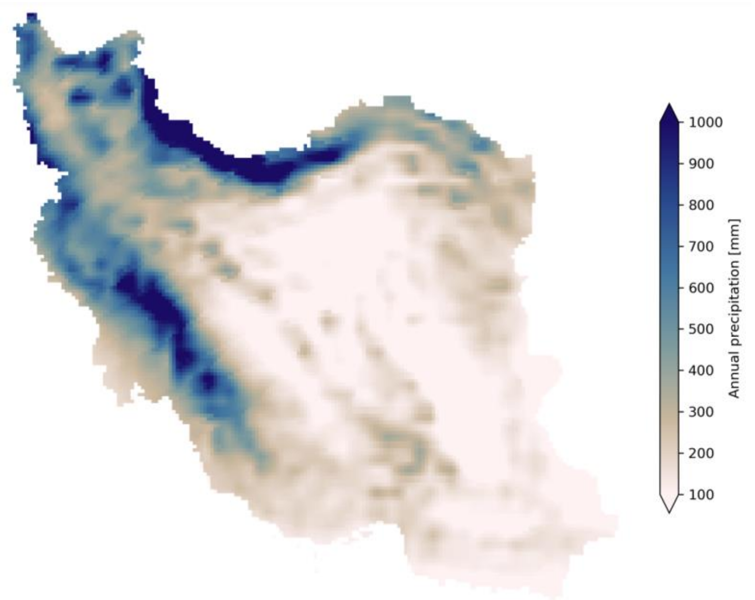

Fig S 3 – Annual average precipitation of Iran. Long-term (1982-2019) average of water years (Oct-Sept) using ERA5-Land. Map was created using Python 3.9 (https://www.python.org/).

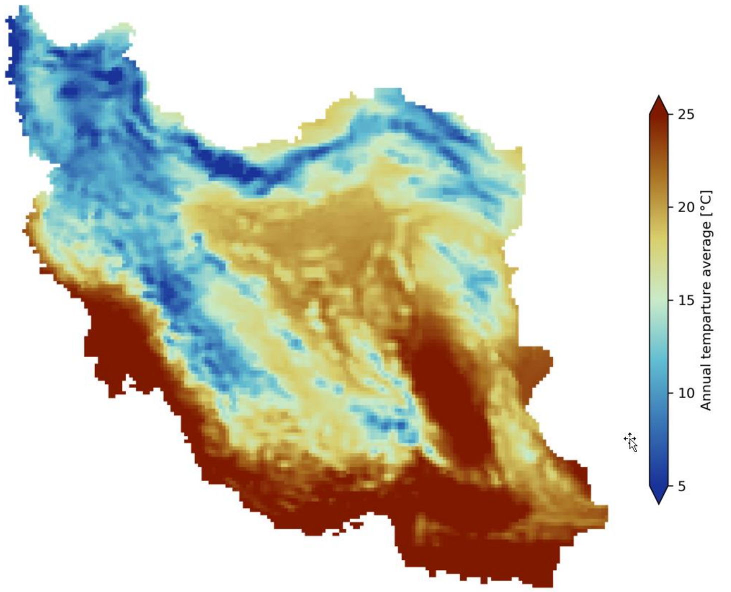

Fig S 4 – Annual average temperature of Iran. Long-term (1982-2019) average of water years (Oct-Sept) using ERA5-Land. Map was created using Python 3.9 (https://www.python.org/).

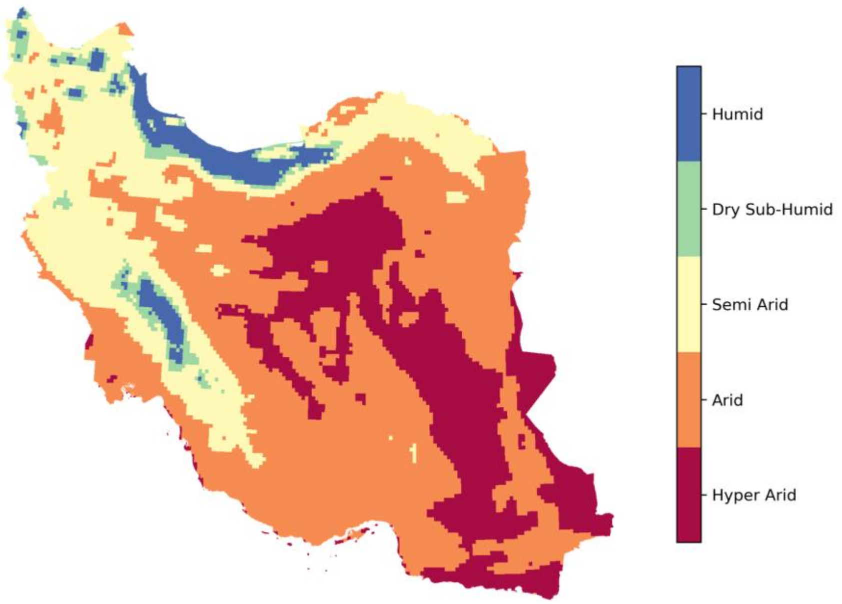

Fig S 5 – Long-term Aridity Index. Derived over the period between 1982 and 2019 using ERA5-Land. Map was created using Python 3.9 (https://www.python.org/).

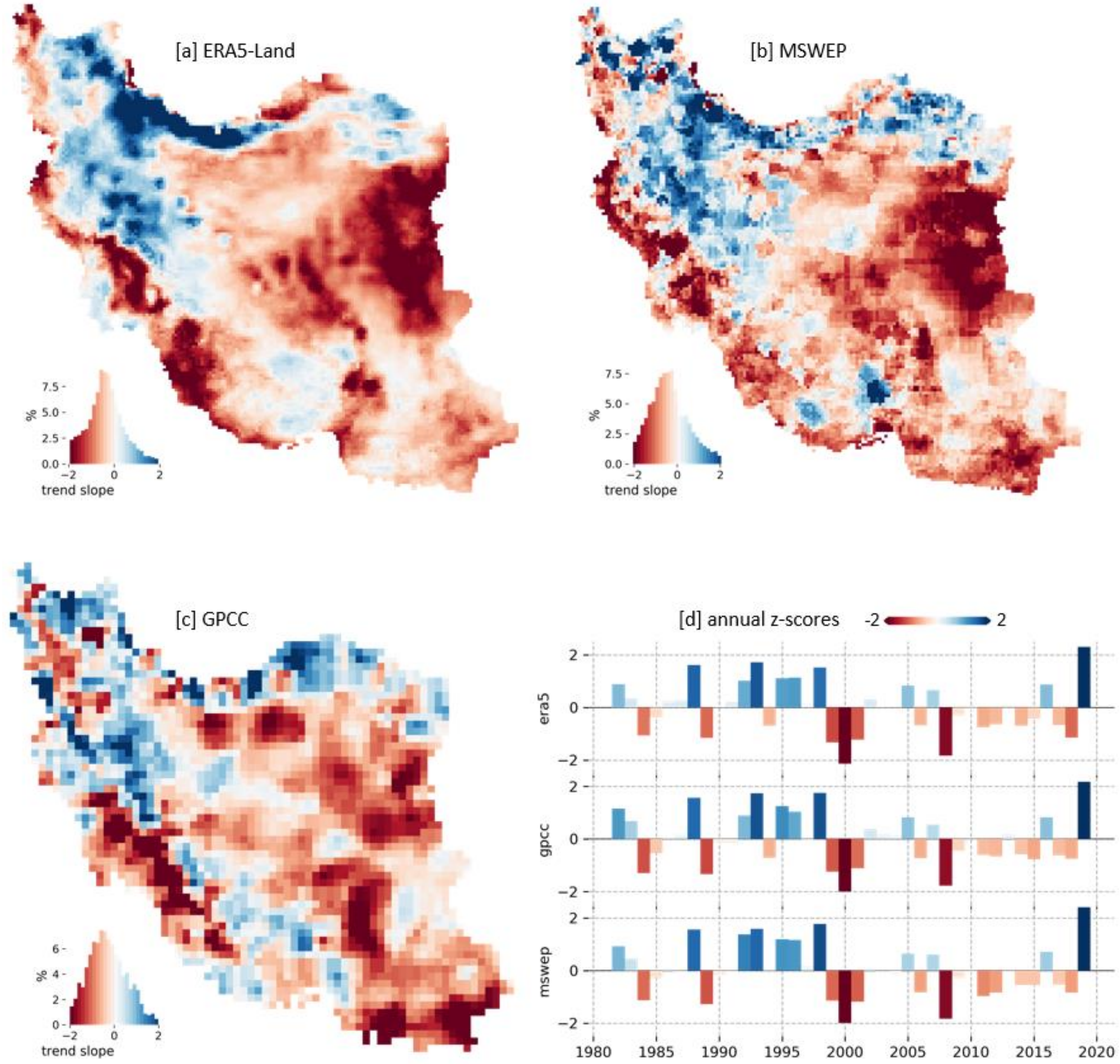

Fig S 6 – Comparison of precipitation products. Pixel-based precipitation trends over Iran for the period 1982 to 2019 in mm per year from ***a:*** *ERA5-Land,* ***b:*** *MSWEP,* ***c:*** *GPCC.* ***d:*** *Iran wide averaged annual variations to the long-term mean given in Z-scores. Note that annual means are calculated for the water year from October 1st to September 30th. Maps were created using Python 3.9 (https://www.python.org/).*

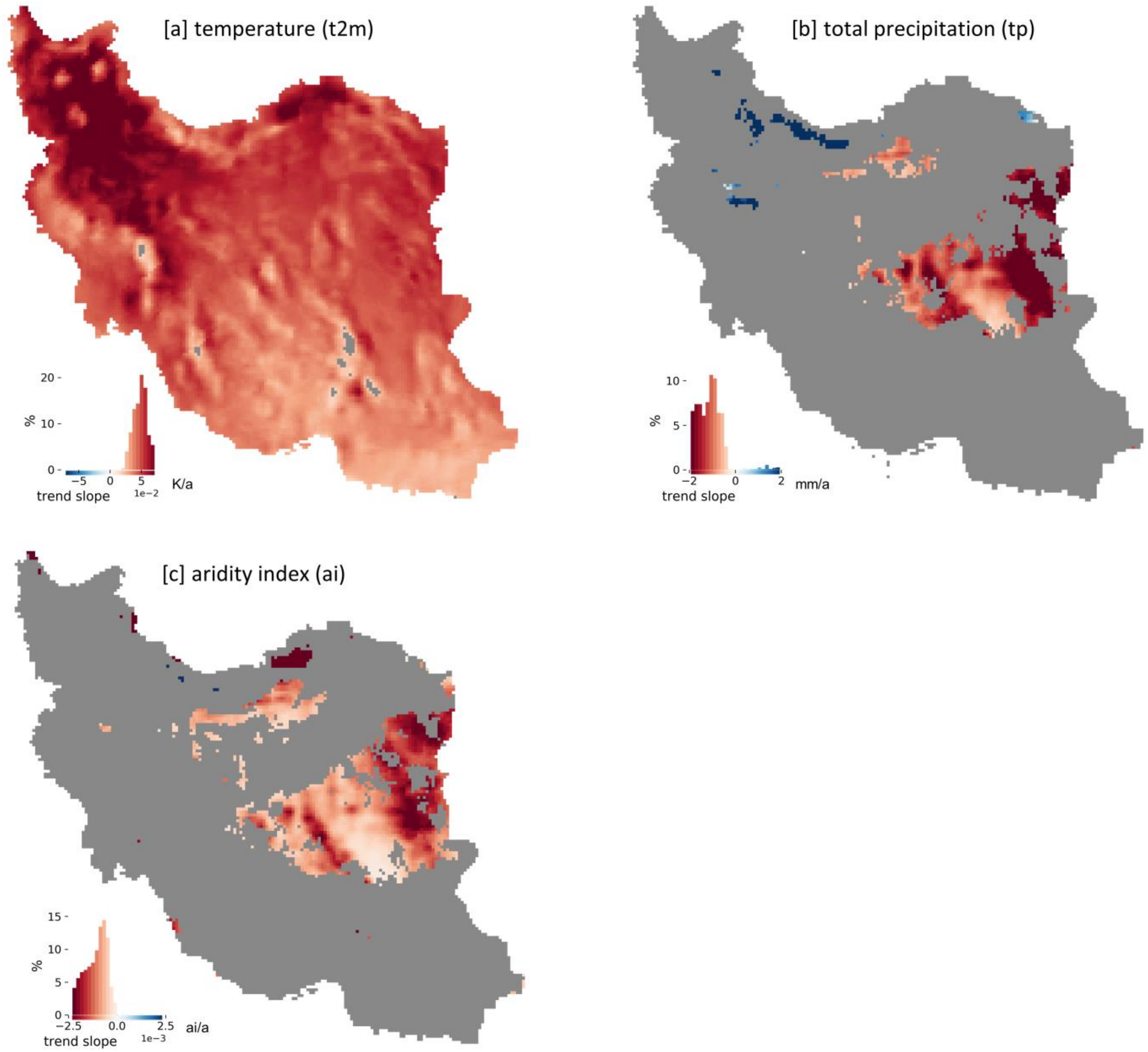

Fig S 7 – Significance of meteorological trends. Derived from ERA5-Land between 1982 and 2019. Trends are considered being statistically significant with a p-value <0.05 using a Mann-Kendall test. Maps were created using Python 3.9 (https://www.python.org/).

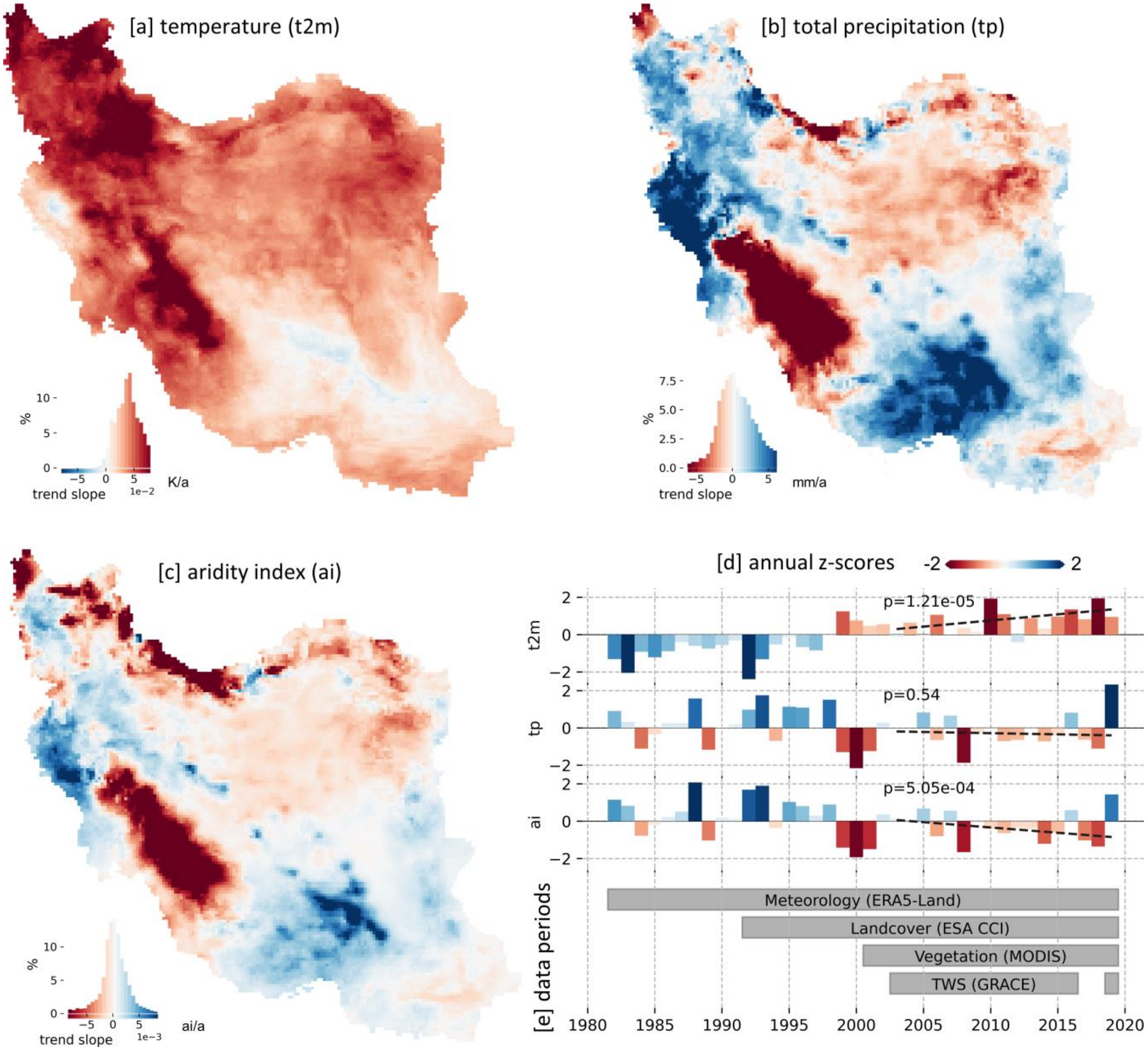

Fig S 8 – Meteorological trends during GRACE(-FO) period. Derived from ERA5-Land between 2003 and 2019. a-c: Trend slope of the gridded data at 0.1 degree. d: Annual variations to the long-term mean (Z-scores of Iran wide averages of the full time period between 1982 and 2019) with trend line (dashed line) along with associated p-values of the trend using Mann-Kendall test for the GRACE(-FO) period. Maps were created using Python 3.9 (https://www.python.org/).

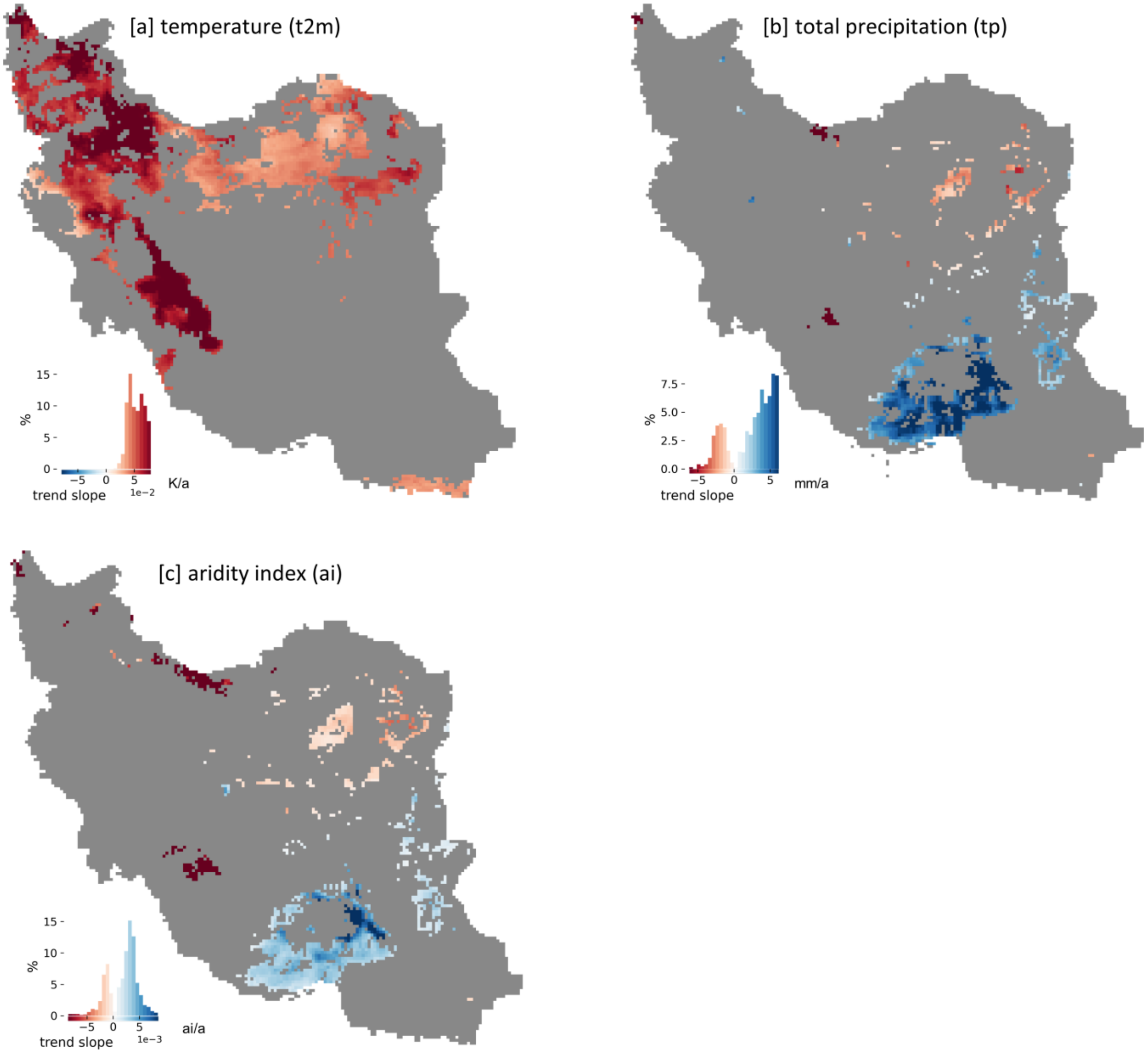

Fig S 9 – Significance of meteorological trends during the GRACE(-FO) period. Derived from ERA5-Land between 2003 and 2019. Trends are considered being statistically significant with a p-value <0.05 using a Mann-Kendall test. Maps were created using Python 3.9 (https://www.python.org/).

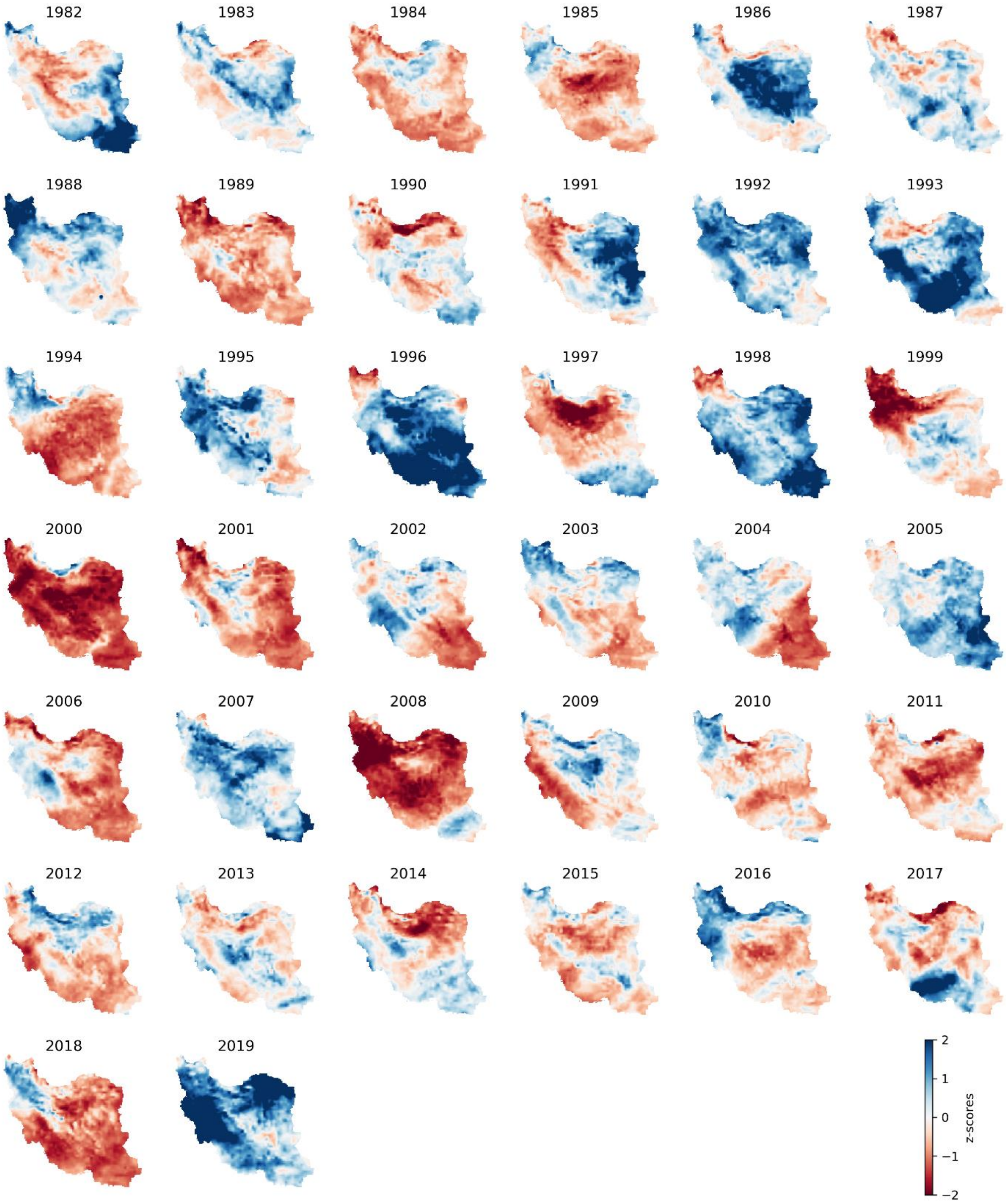

Fig S 10 – Annual Z-score maps of ERA5-Land total precipitation (tp) in Iran between 1982 and 2019. Calculated for water years (Oct-Sept). Long-term baseline for Z-score derivation uses the full time period 1982-2019. Maps were created using Python 3.9 (https://www.python.org/).

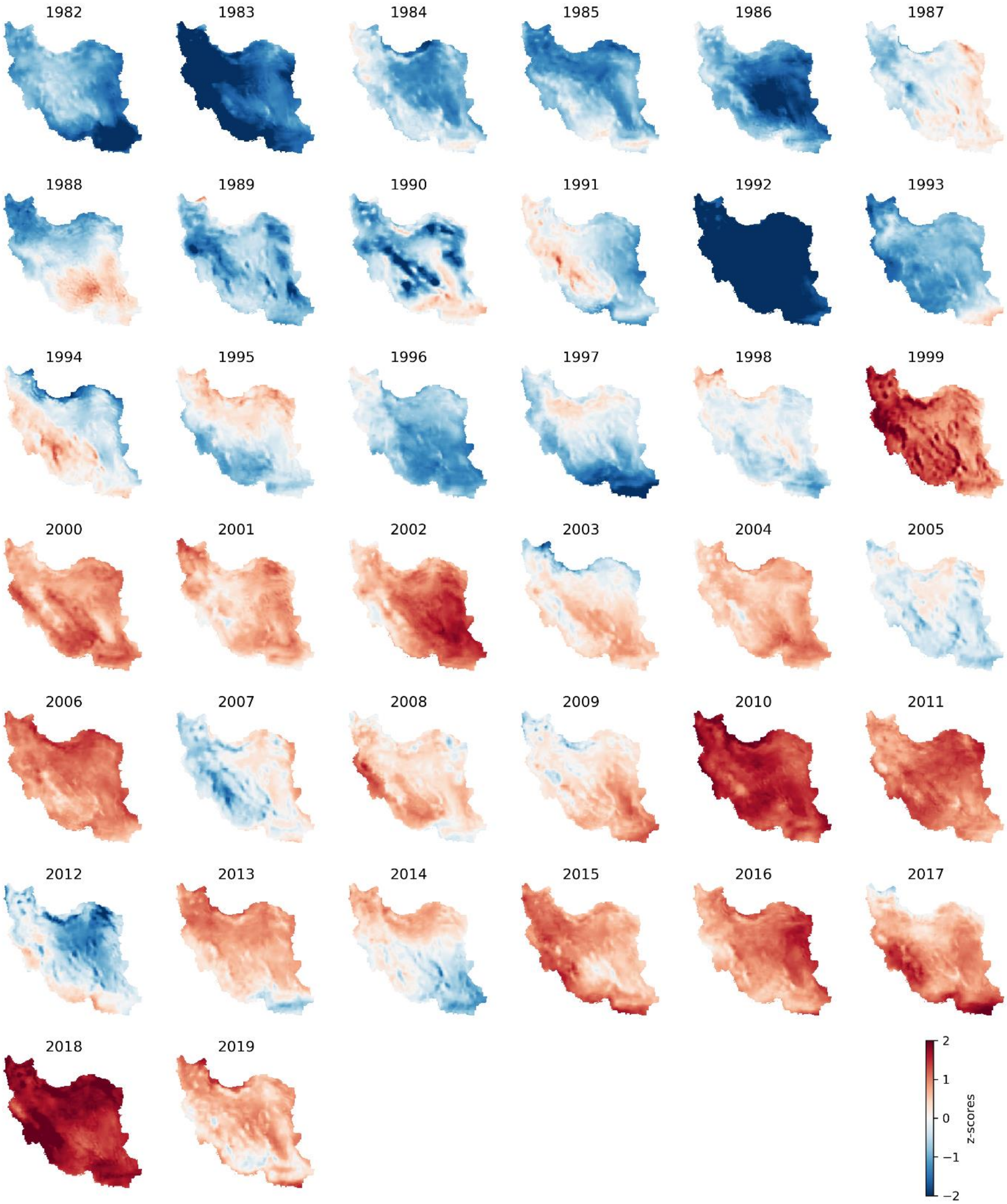

Fig S 11 – Annual Z-score maps of ERA5-Land temperature (t2m) in Iran between 1982 and 2019. Calculated for water years (Oct-Sept). Long-term baseline for Z-score derivation uses the full time period 1982-2019. Maps were created using Python 3.9 (https://www.python.org/).

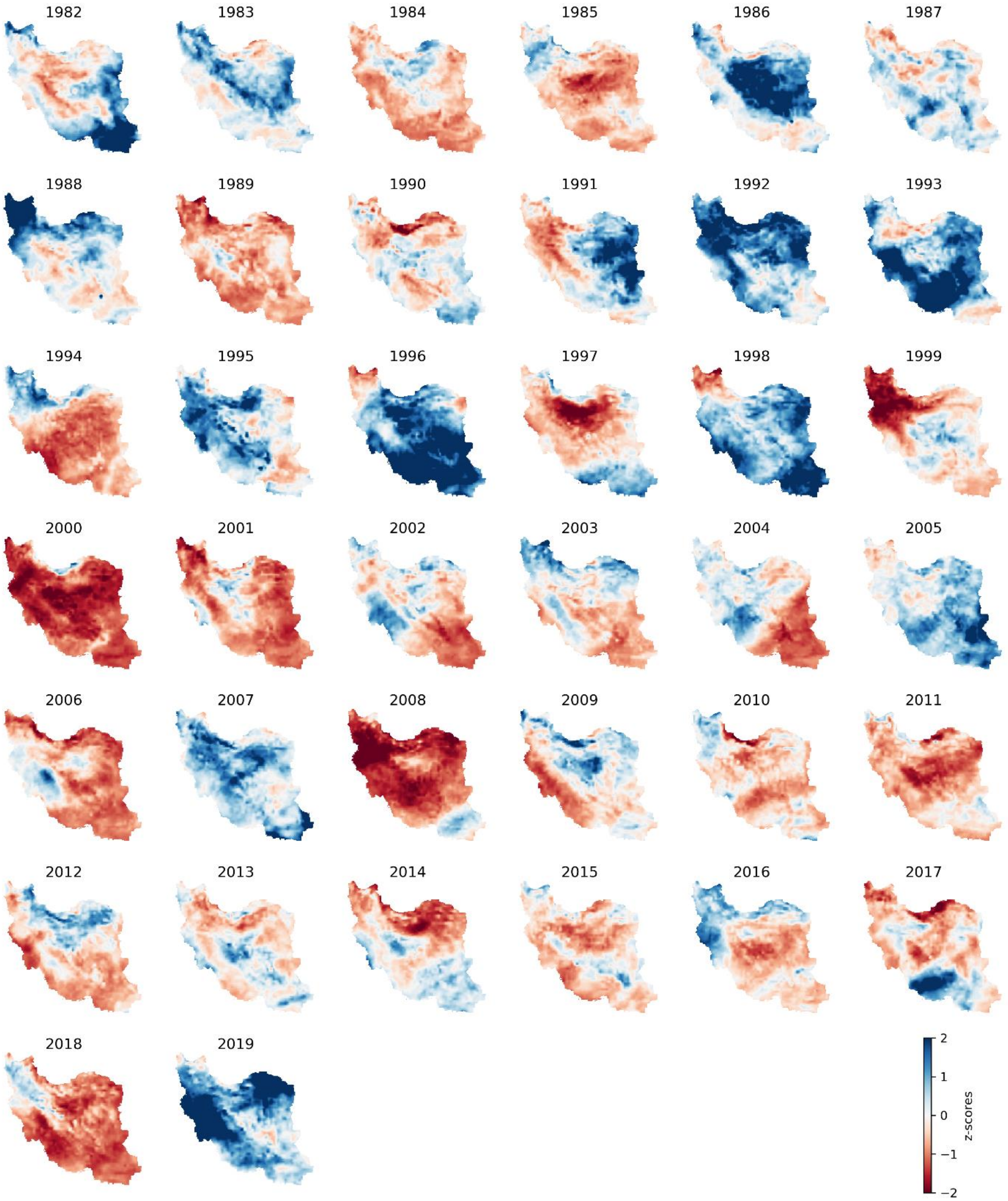

Fig S 12 – Annual Z-score maps of ERA5-Land aridity index (ai) in Iran between 1982 and 2019. Calculated for water years (Oct-Sept). Long-term baseline for Z-score derivation uses the full time period 1982-2019. Maps were created using Python 3.9 (https://www.python.org/).

S.3 Land cover

Table S 1 – Reclassification scheme of the ESA CCI land cover product into the classes and color codes used throughout the manuscript.

manuscript		ESA CCI land cover	
color	class	code	Description
	crops	10	Cropland, rainfed
		11	Cropland, rainfed, herbaceous cover
		12	Cropland, rainfed, tree or shrub cover
		20	Cropland, irrigated or post-flooding
		30	Mosaic cropland (>50%) / natural vegetation (tree, shrub, herbaceous cover) (<50%)
		40	Mosaic natural vegetation (tree, shrub, herbaceous cover) (>50%) / cropland (<50%)
	trees	50	Tree cover, broadleaved, evergreen, closed to open (>15%)
		60	Tree cover, broadleaved, deciduous, closed to open (>15%)
		61	Tree cover, broadleaved, deciduous, closed (>40%)
		62	Tree cover, broadleaved, deciduous, open (15-40%)
		70	Tree cover, needleleaved, evergreen, closed to open (>15%)
		71	Tree cover, needleleaved, evergreen, closed (>40%)
		72	Tree cover, needleleaved, evergreen, open (15-40%)
		80	Tree cover, needleleaved, deciduous, closed to open (>15%)
		81	Tree cover, needleleaved, deciduous, closed (>40%)
		82	Tree cover, needleleaved, deciduous, open (15-40%)
		90	Tree cover, mixed leaf type (broadleaved and needleleaved)
		100	Mosaic tree and shrub (>50%) / herbaceous cover (<50%)
	sparse	110	Mosaic herbaceous cover (>50%) / tree and shrub (<50%)
		120	Shrubland
		121	Evergreen shrubland
		122	Deciduous shrubland
		130	Grassland
		140	Lichens and mosses
		150	Sparse vegetation (tree, shrub, herbaceous cover) (<15%)
		151	Sparse tree (<15%)
		152	Sparse shrub (<15%)
		153	Sparse herbaceous cover (<15%)
	inundated	160	Tree cover, flooded, fresh, or brackish water
		170	Tree cover, flooded, saline water
		180	Shrub or herbaceous cover, flooded, fresh/saline/brackish water
	urban	190	Urban areas
	bare	200	Bare areas
		201	Consolidated bare areas
		202	Unconsolidated bare areas
	water	210	Water bodies
		220	Permanent snow and ice

Table S 2 – Land cover change between 1992 and 2019 using reclassified ESA CCI land cover classes Table S 1

Land cover	1992 [km²]	2019 [km²]	change [km²]	change [%]
crops	292447	319218	26771	9
trees	21916	19606	-2310	-11
sparse	264733	215488	-49245	-19
inundated	855	1008	153	18
urban	2427	6464	4037	166
bare	1036619	1060652	24033	2
water	10843	7404	-3439	-32

S.4 Annual vegetation growth $NDVI_{MEAN*}$ ancillary data

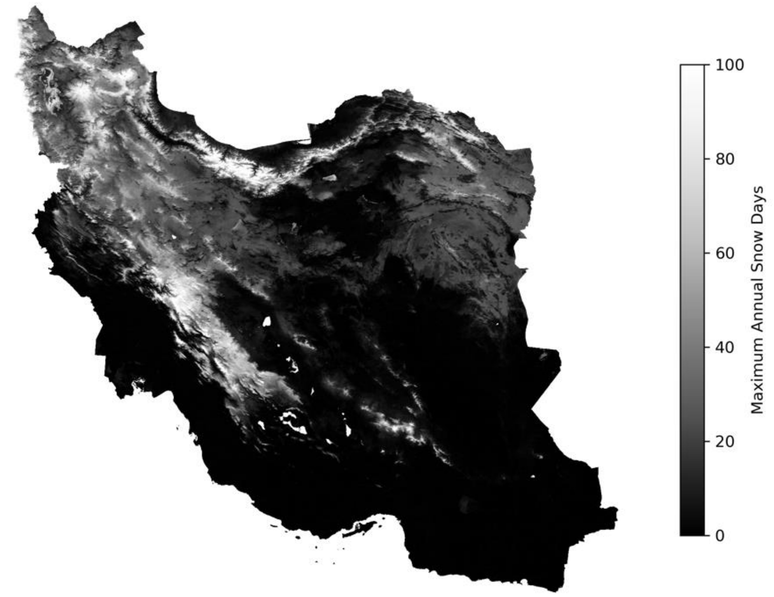

Fig S 13 – $MaxSnowDays$. Long-term (2001-2019) maximum days of snow cover during a water year (Oct-Sept) derived by MODIS Snow cover product (MOD10A1 V6). Input for the annual vegetation (NDVI) aggregate used in this study (Methods). Note: In some dried wetlands/saltpans unusual high values occur due to misinterpretation of the spectral signal in the algorithm to produce the MODIS snow cover product. Those areas are not affecting the results of the study, because these non-vegetated areas are not part of the analysis. Map was created using Python 3.9 (https://www.python.org/).

S.5 Differentiation of agricultural and natural vegetation ancillary data

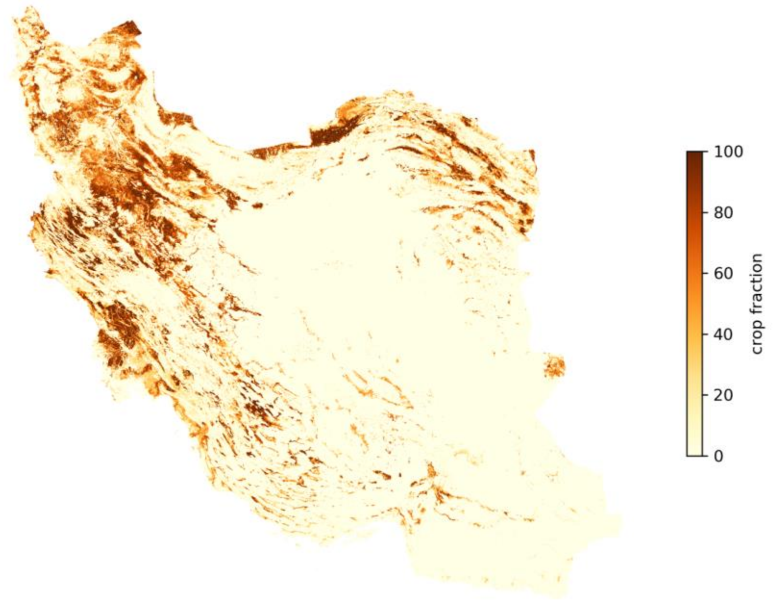

Fig S 14 - Crop fraction per MODIS pixel. Calculated from the maximum crop fraction between 2015 and 2019 using the 100m crop fraction layer of the Copernicus Global Land Service land cover product (CGLS -LC100). An averaging interpolation technique used to resample to MODIS 250m pixel size. Map was created using Python 3.9 (https://www.python.org/).

S.6 Trends and annual dynamics of vegetation

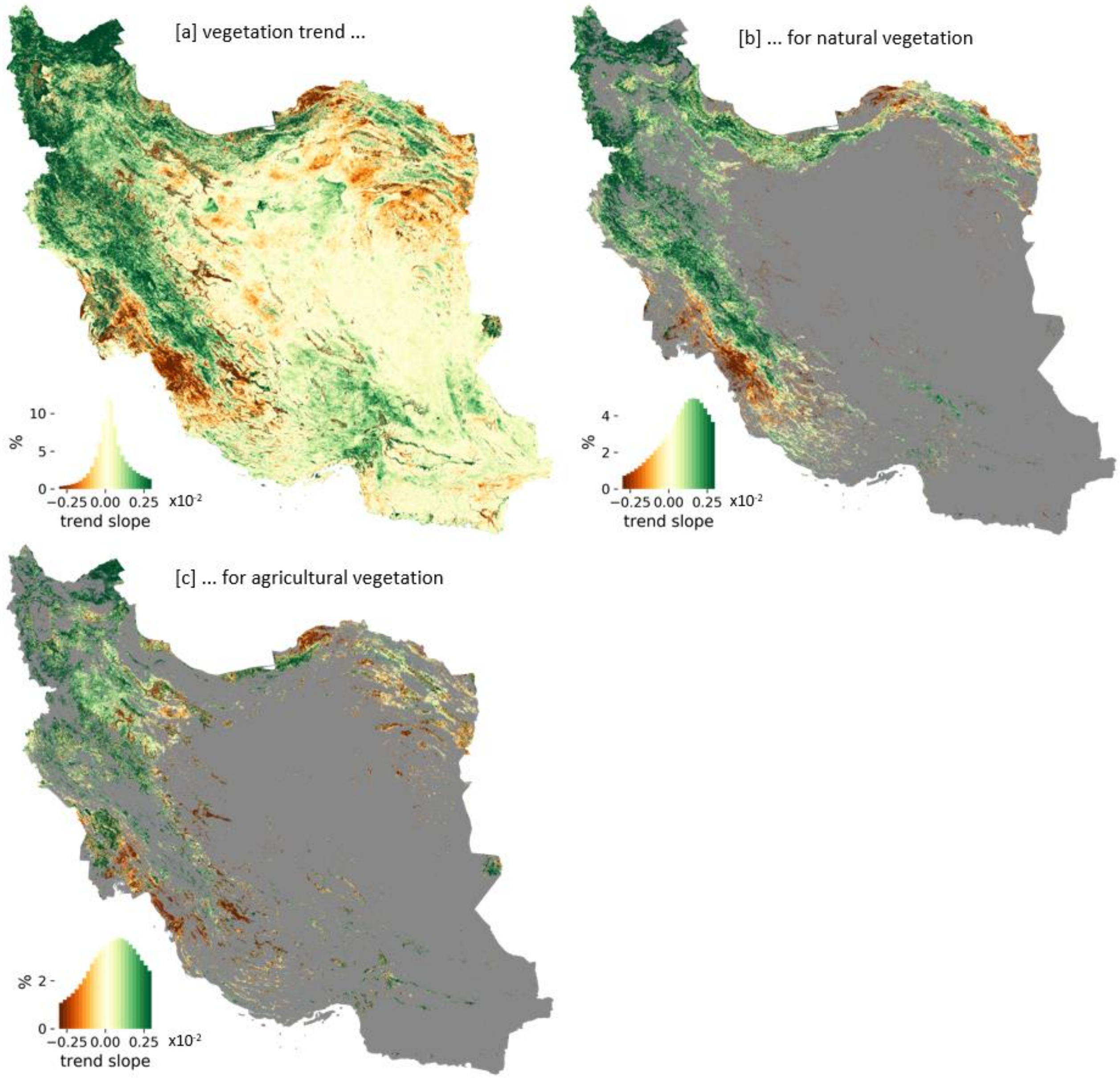

Fig S 15 – Vegetation trend between 2005 and 2018. It excludes the very dry period at the beginning (until 2001, see Figure 1) including the subsequent year, in which vegetation is still influenced by the intense drought conditions (2002-2004, see Fig S4) and the wet year at the end (2019) of the analyzed vegetation analysis. Compared to the complete period 2001-2019 (Figure 5), the spatial patterns of vegetation trends remain very similar. Maps were created using Python 3.9 (https://www.python.org/).

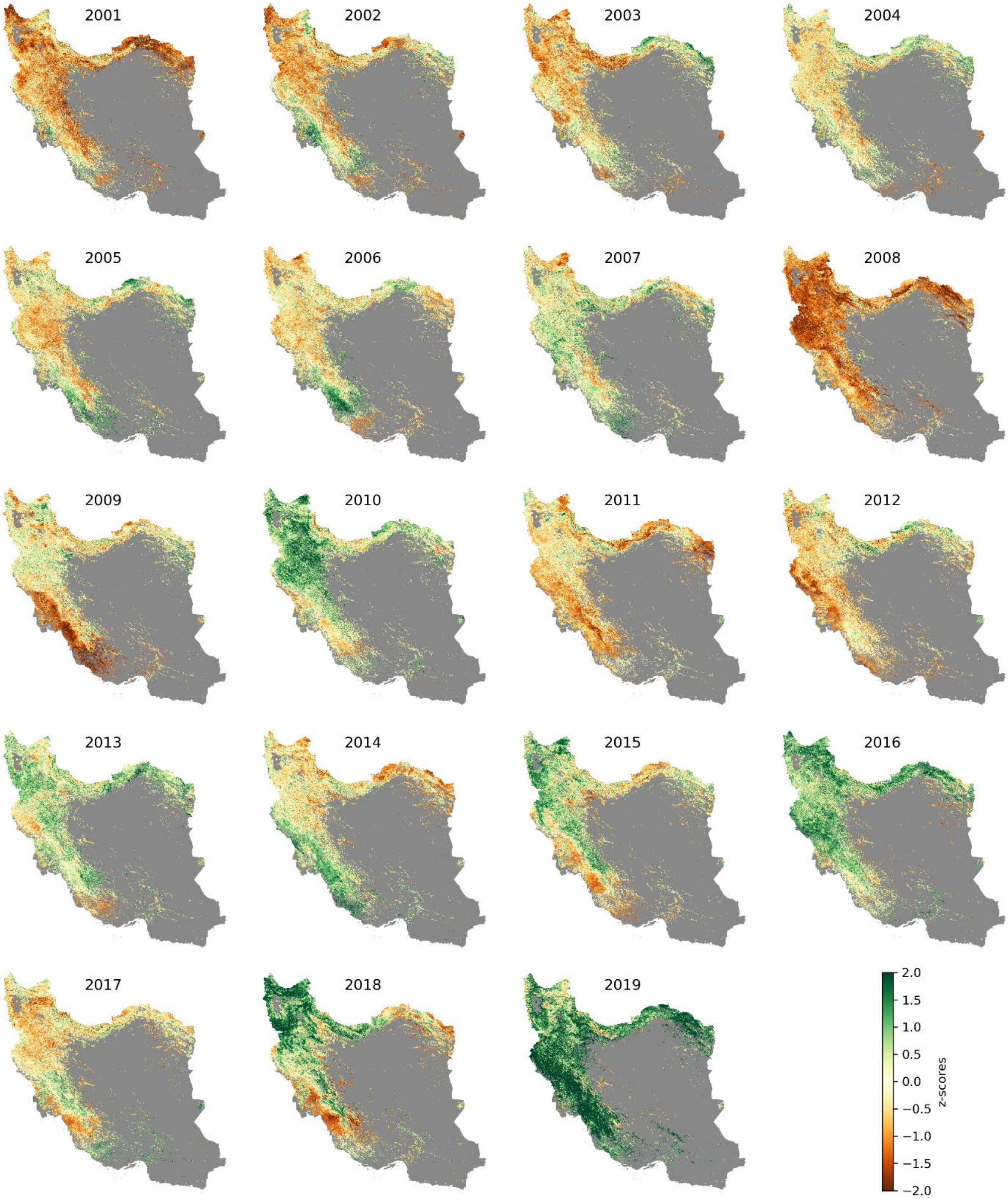

Fig S 16 – Annual Z-score maps of vegetated pixels (both, natural and agricultural vegetation) in Iran. Calculated for water years (Oct-Sept). Long-term baseline for Z-score derivation uses the full time period 2001-2019. Maps were created using Python 3.9 (https://www.python.org/).

S.7 Natural vegetation compared to meteorological conditions

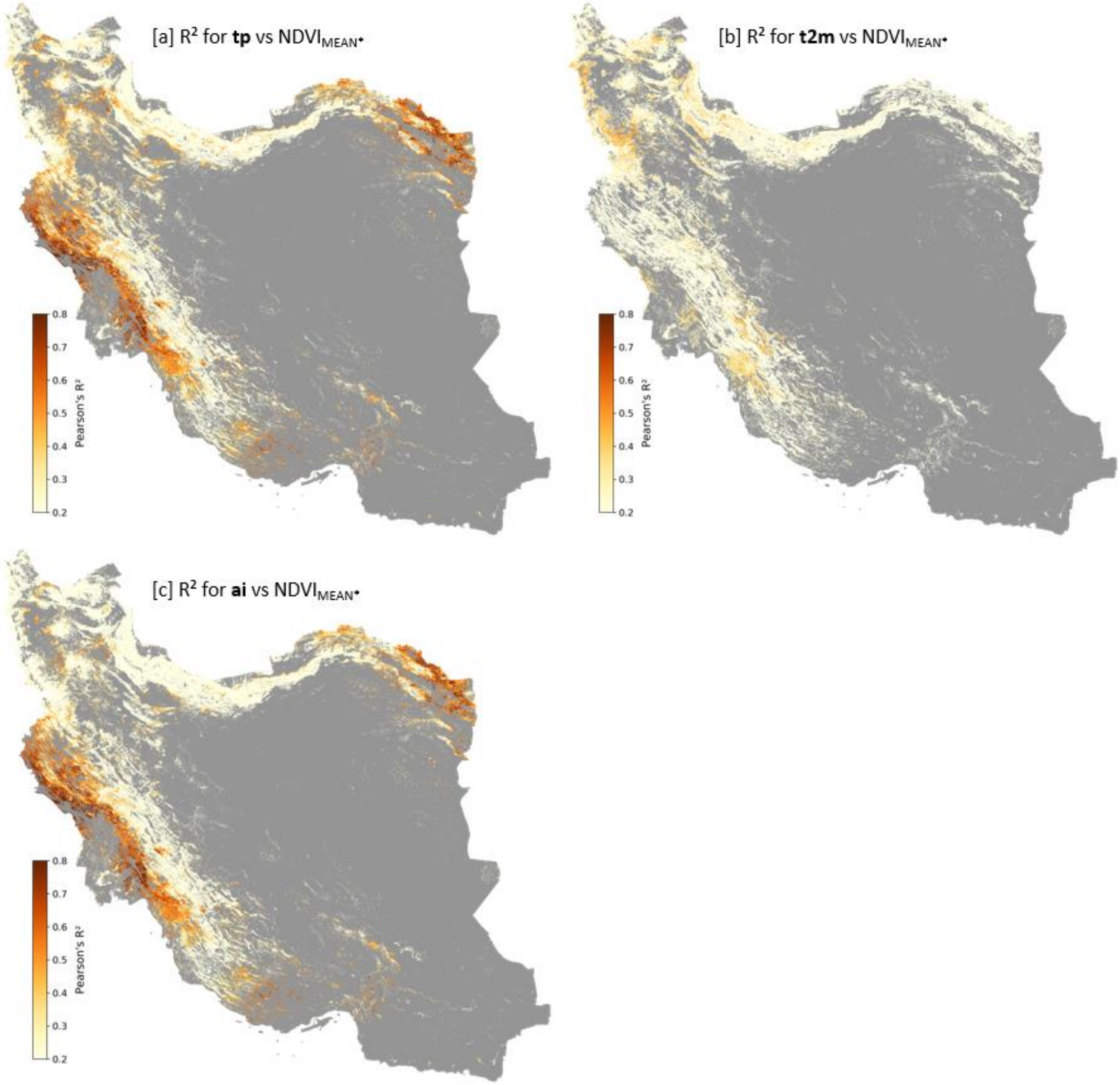

Fig S 17 – Natural vegetation dynamics against meteorological conditions. Pearson correlation of annual vegetation growth ($NDVI_{MEAN*}$) and annual aggregates of meteorological parameters. ***a:*** *total precipitation (tp),* ***b:*** *temperature (t2m),* ***c:*** *aridity index (ai)***.** *Maps were created using Python 3.9 (https://www.python.org/).*

S.8 Agricultural vegetation compared to hydrometeorology

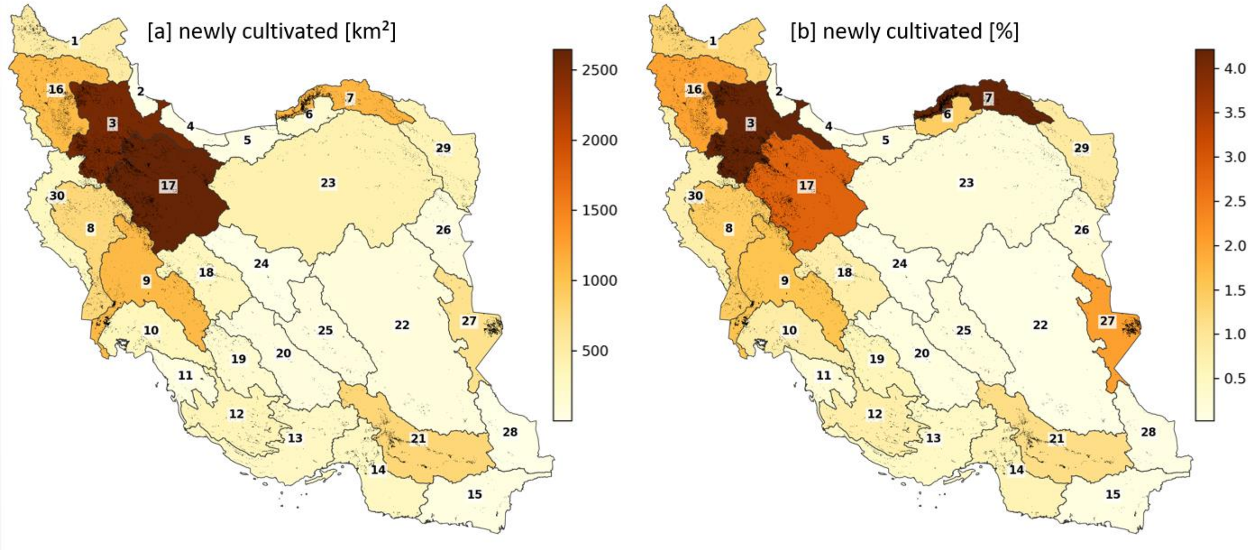

*Fig S 18 – Newly cultivated areas per basin in km² (**a**) and percentage (**b**). For the definition of the term "newly cultivated" see methods. Note: Pixels defined as newly cultivated are plotted in black on top of the basin map. Maps were created using Python 3.9 (https://www.python.org/).*

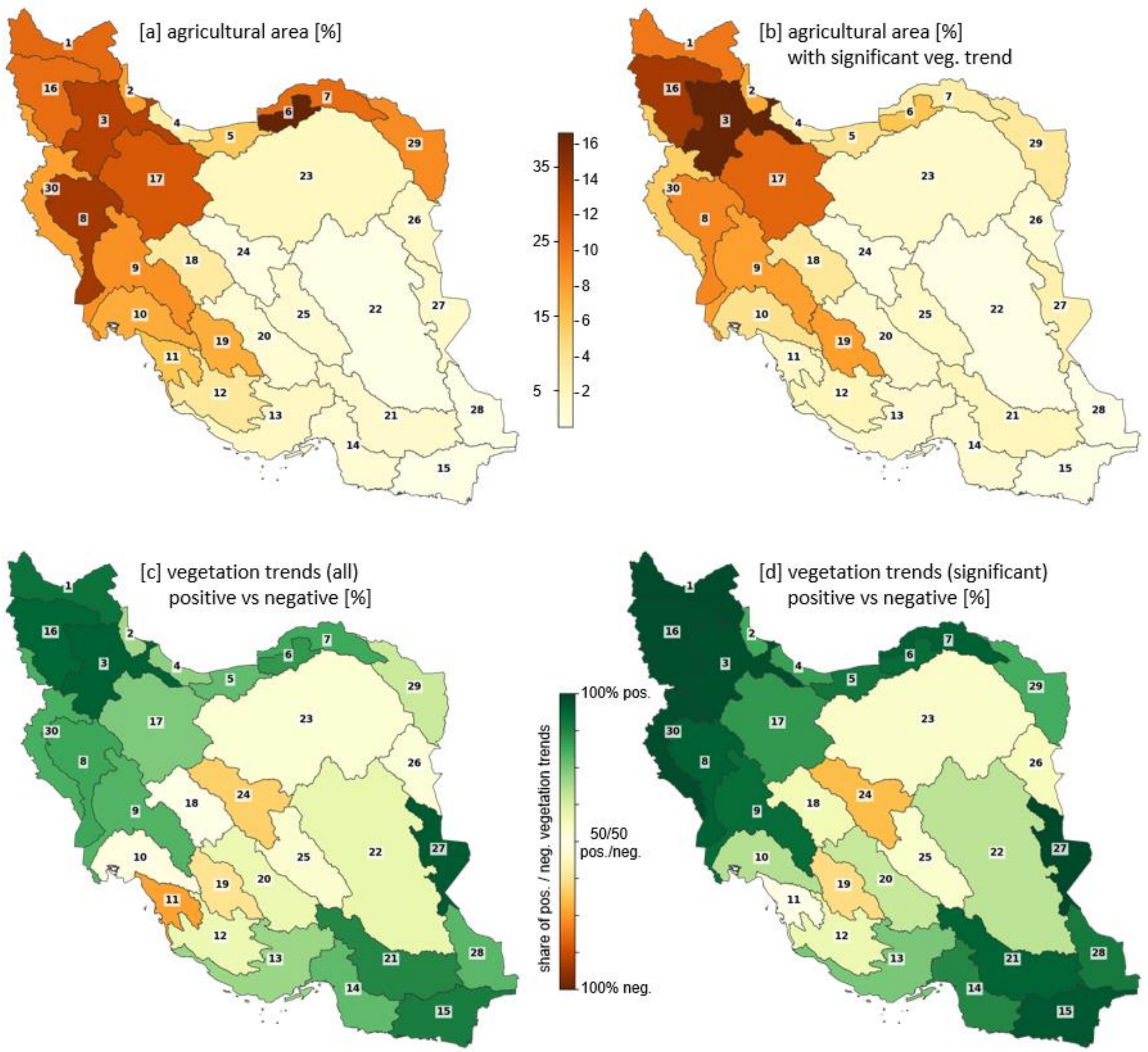

Fig S 19 – Agricultural areas and associated vegetation trends per basin. ***a:*** *Agricultural area per basin. Each pixel's area is normalized by its crop fraction (pixel area * crop fraction), whereas only pixels with a crop fraction exceeding 20% are considered as agricultural pixels.* ***b:*** *Agricultural area with a significant vegetation trend between 2001 and 2019 (p-value <0.05) per basin.* ***c:*** *Proportion of positive and negative vegetation trends considering all agricultural pixels.* ***d:*** *Proportion of positive and negative vegetation trends considering only pixels with significant trends. General note: agricultural areas which were newly cultivated during the analyzed time span are neglected (see methods). Maps were created using Python 3.9 (https://www.python.org/).*

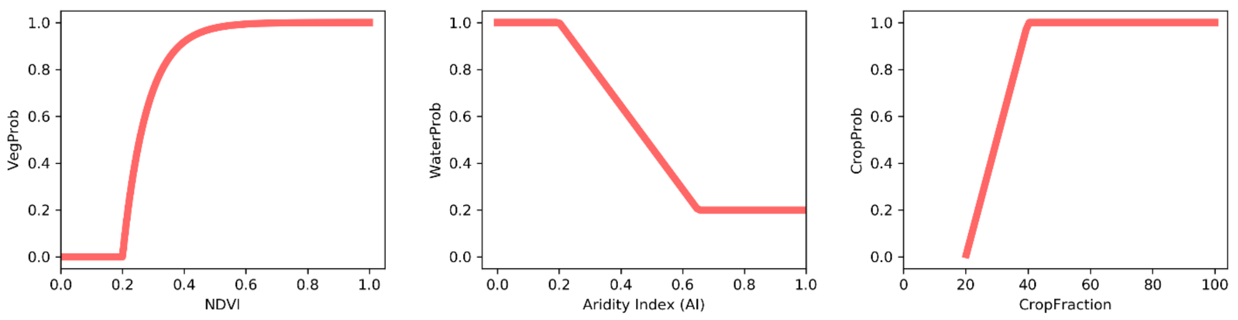

Fig S 20 – Visualization of the calculation rules for VegProb, WaterProb, and CropProb. (see methods for further details).

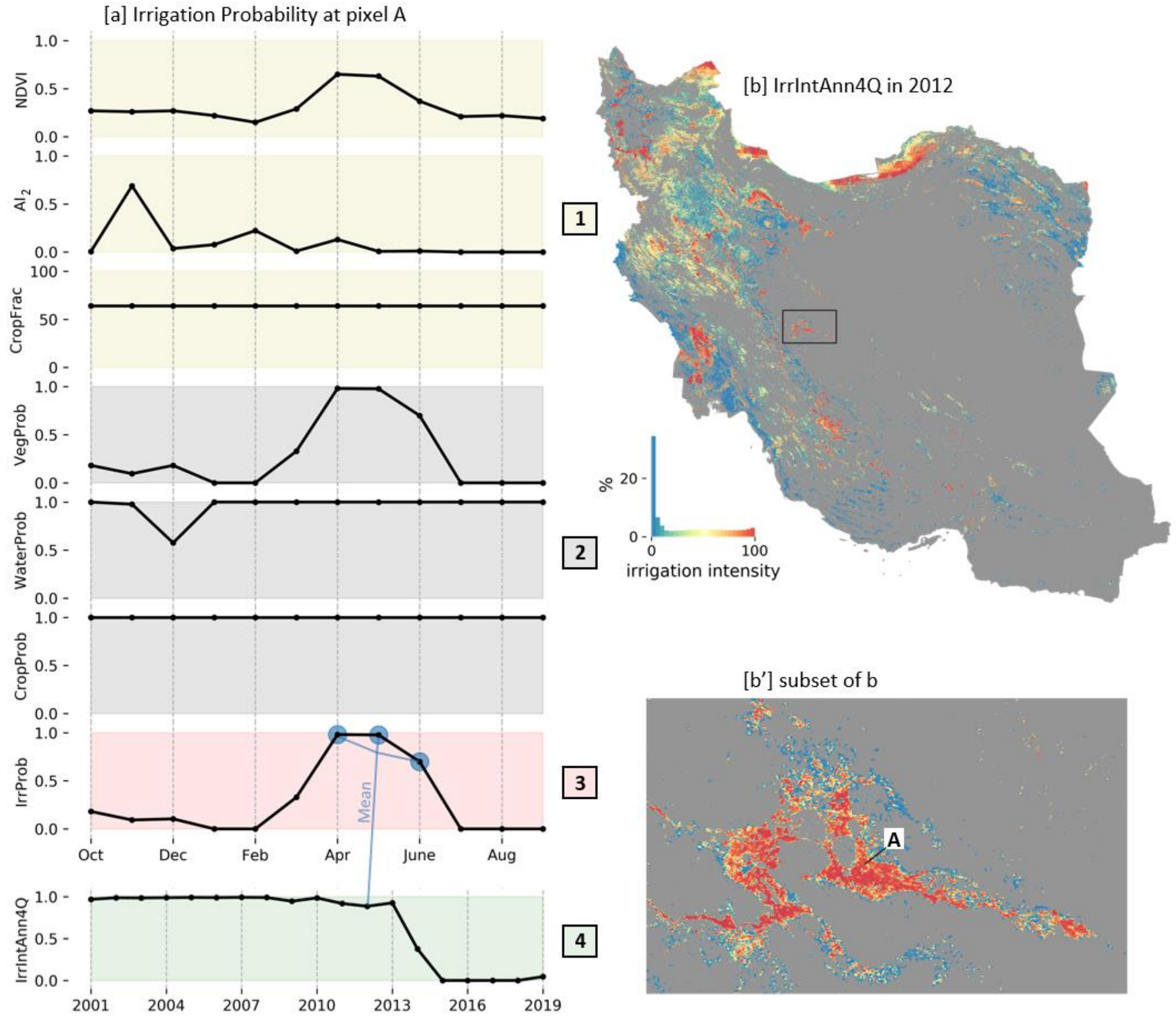

Fig S 21 – Irrigation probability derivation. ***a:*** *At example pixel A (same pixel as in Figure 5) the derivation of monthly irrigation probability (IrrProb) and annual irrigation intensity (IrrIntAnn4Q) is shown.* ***1****: Input parameter at pixel A in the water year 2012 (Oct 2011 – Sept 2012). NDVI: monthly MODIS NDVI composites; AI_2: ERA5-Land monthly aridity index (ai) calculated over 2 subsequent months (previous and current month); CropFrac: Maximum crop fraction between 2015 and 2019 (see Fig S 14). Note: CropFrac is a single value per pixel and thus is not changing over time.* ***2****: Monthly VegProb, WaterProb, and CropProb. Derived from NDVI, AI_2, and CropFrac, respectively. (For calculation details, please see Methods and Fig S 20).* ***3:*** *Calculation of monthly irrigation probability (IrrProb). IrrProb = VegProb * WaterProb * CropProb.* ***4:*** *Annual irrigation intensity (IrrIntAnn4Q). IrrIntAnn4Q represents the mean of the 4th Quantile of monthly IrrProb values during the year.* ***b:*** *Map of IrrIntAnn4Q for Iran for the water year 2012.* ***b’:*** *subset of b, showing the agricultural areas around Isfahan. Map was created using Python 3.9 (https://www.python.org/).*

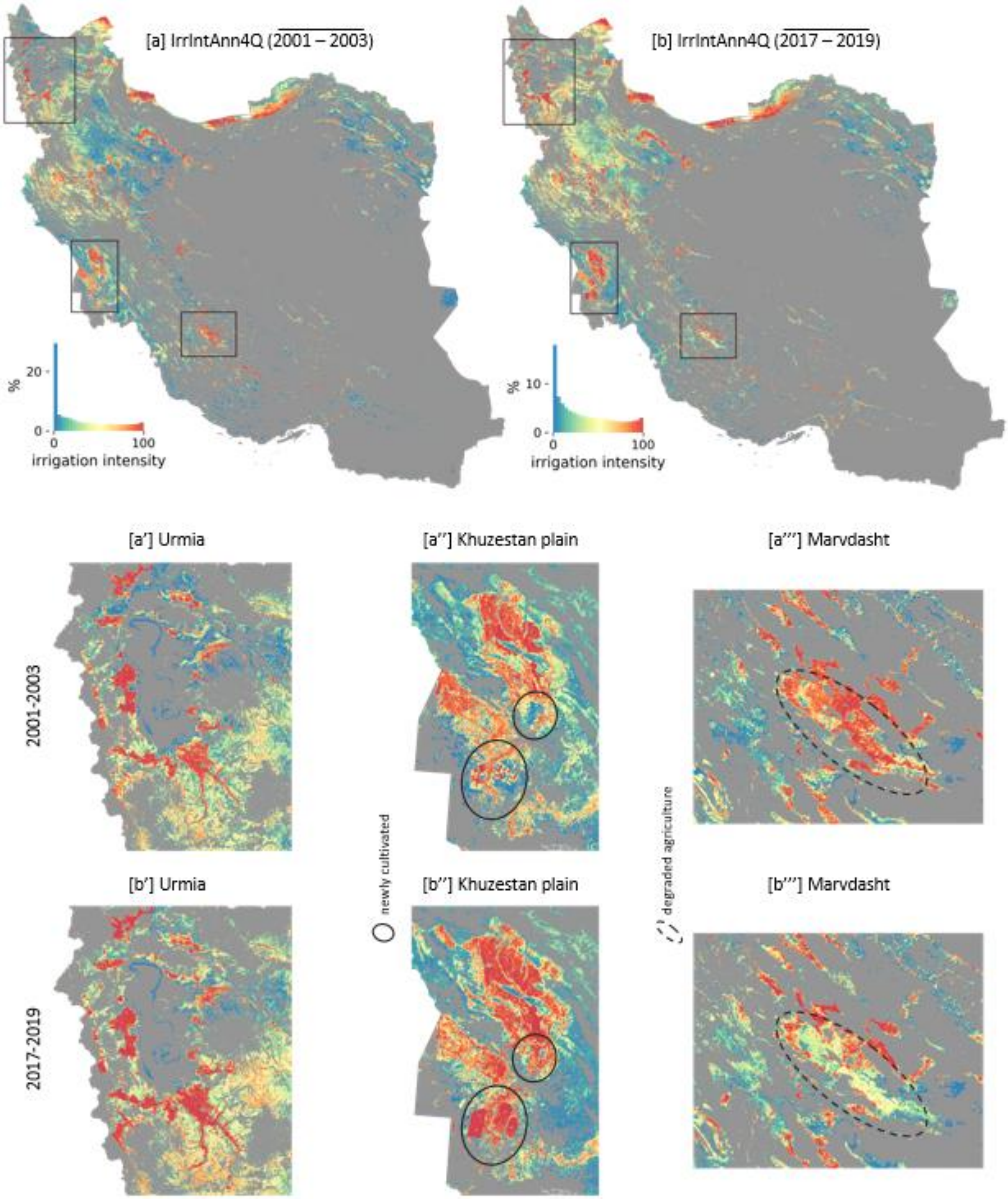

*Fig S 22 – Comparison of irrigation intensities (3 year averages of IrrIntAnn4Q) from 2001-2003 (**a**) and 2017-2019 (**b**). Maps were created using Python 3.9 (https://www.python.org/).*

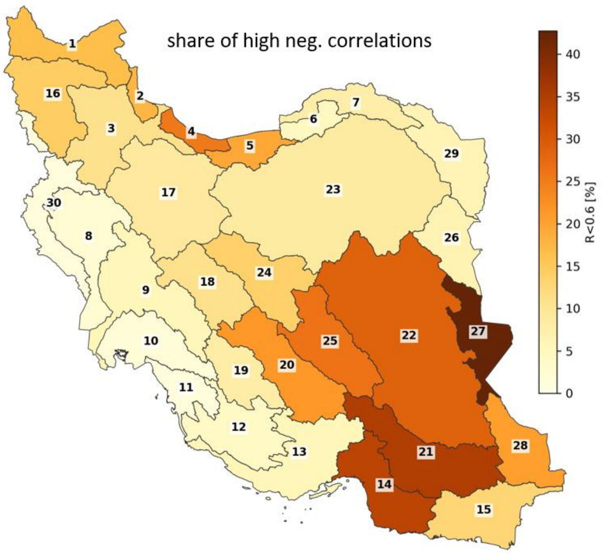

Fig S 23 – Share of high negative correlations (R<-0.6) between annual vegetation growth ($NDVI_{MEAN*}$) and annual total water storage anomalies (TWSA) for agricultural areas. Map was created using Python 3.9 (https://www.python.org/).
